# Understanding strain localization in metallic materials: a review of high-resolution digital image correlation and related techniques

**DOI:** 10.1080/14686996.2026.2630488

**Published:** 2026-02-17

**Authors:** F. Briffod, T. E. J. Edwards, J. Quinta da Fonseca, J. -C. Stinville, D. Texier, T. Vermeij

**Affiliations:** a Research Center for Structural Materials, National Institute for Materials Science, Tsukuba, Japan; b Department of Materials Science, Institute of Pure and Applied Sciences, University of Tsukuba, Tsukuba, Japan; c Department of Materials, The University of Manchester, Manchester, UK; d Materials Science and Engineering Department, University of Illinois at Urbana-Champaign, Urbana, IL, USA; e Univ Toulouse, IMT Mines Albi, INSA Toulouse, ISAE-SUPAERO, CNRS, ICA, Albi, France; f Laboratory for Mechanics of Materials and Nanostructures, Empa Swiss Federal Laboratories for Material Science and Technology, Thun, Bern, Switzerland

**Keywords:** High-resolution digital image correlation, strain localization, crystal plasticity, data merging, metallic materials, machine learning, SEM-DIC, HR-DIC

## Abstract

Plastic deformation in metallic materials is generally governed by highly localized and intrinsically heterogeneous deformation processes, including crystallographic slip banding, deformation twinning, phase transformation and grain-boundary sliding. These mechanisms operate at the sub-grain scale where they are competing, interacting, and are sometimes incompatible for short-range transmission due to deformation confinement within individual grains. The heterogeneous nature of irreversible deformation at the microstructure scale also applies at the mesoscale, *i.e*. the scale of the crystalline aggregate. Capturing experimentally the discrete and heterogeneous deformation processes at the microstructure scale is essential to understand elementary deformation processes involved for specific loading conditions, quantifying their intensity to finally achieve a better dialogue with numerical models of crystal plasticity for the prediction of mechanical behavior and the lifetime of parts. High-resolution digital image correlation (HR-DIC), implemented on scanning electron microscopy images, has emerged as a key technique to quantify these phenomena by providing full-field measurements of in-plane displacement and strain at sub-micron spatial resolution over statistically representative fields of view. This review outlines the experimental foundations, data-processing strategies, and correlative analysis frameworks that underpin the use of HR-DIC for studying strain localization in metals.

## IMPACT STATEMENT

This review synthesizes advances in high-resolution digital image correlation, highlighting its integration with microstructure, crystal plasticity, and machine learning to reveal strain localization mechanisms in metals.

## GRAPHICAL ABSTRACT

**Figure:**
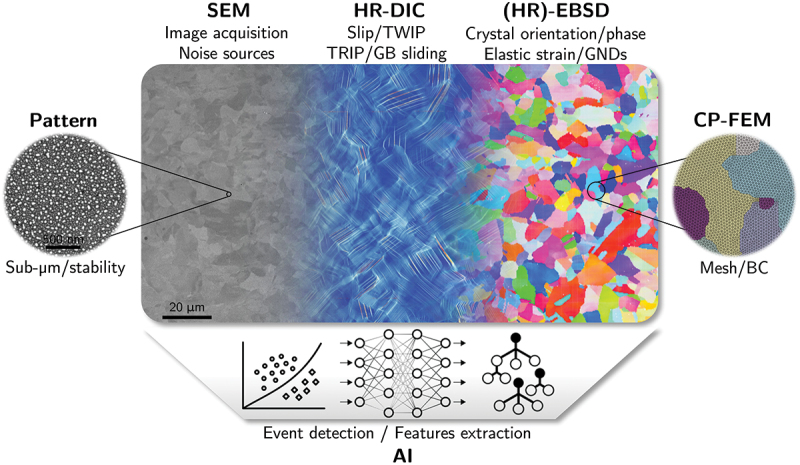

## 1. Introduction

### 1.1. Strain localization in metallic materials

Plastic deformation in metallic materials is inherently heterogeneous, driven by the collective motion of dislocations and their complex interactions with microstructural features such as grain boundaries, precipitates, inclusions or phase interface. This heterogeneity is further exacerbated by the intrinsically multi-scale and polycrystalline nature of metals, where plasticity initiates at the nanoscopic scale through dislocation activity and evolves at the mesoscopic scale across grains and grain clusters up to the macroscopic scale. These localized deformation processes manifest as slip bands, Lüders bands, deformation twins, phase transformation, or shear bands, which govern the onset of plasticity, the plastic instability, the damage initiation, and eventual fracture [1–7]. Characterizing these localization phenomena is therefore essential for understanding and modeling the mechanical behavior of metals across multiple length scales.

Traditionally, deformation characterization of materials has relied on the combination of macroscopic mechanical tests, providing global stress-strain responses, and microstructural analyses performed before and after deformation using optical microscopy (OM), scanning electron microscopy (SEM), or transmission electron microscopy (TEM). While TEM has long been the primary tool for probing deformation mechanisms at the nanoscale, it provides only highly local information within thin foils and typically captures a static snapshot of the microstructure after dislocation activity has occurred. Consequently, TEM excels at resolving dislocation configurations and defect interactions but cannot directly capture the continuous spatio-temporal evolution of strain localization during *in-situ* deformation over statistically representative regions. Bridging the gap between local, high-resolution characterization and full-field quantitative deformation mapping has been the driving motivation behind the development of high-resolution digital image correlation (HR-DIC).

### 1.2. Brief history of DIC

**Figure 1.**
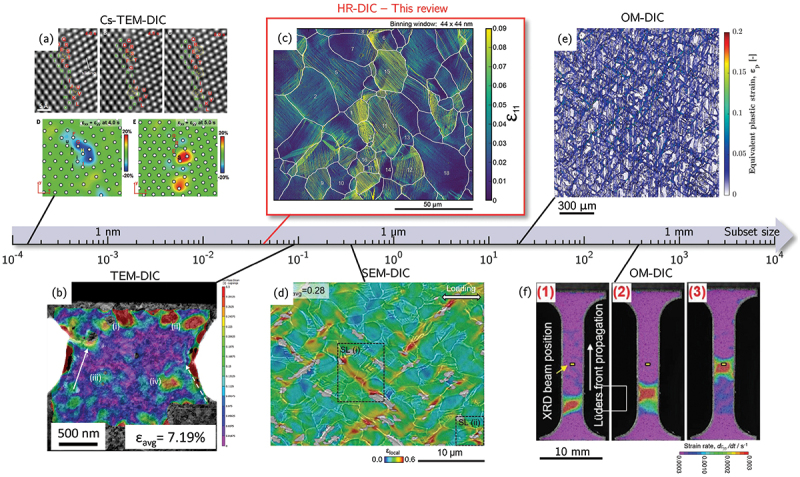
Examples of application of DIC using different observation means and associated scales. The central scalebar represents the achievable subset size. (a) Grain boundary sliding and corresponding estimated strain fields of a platinum bicrystal by aberration-corrected transmission electron microscope (Cs-TEM) (adapted from [12] with permission from AAAS). (b) Strain field on the surface of a nanocrystalline copper tensile specimen during *in-situ* tensile test inside a TEM (adapted from [13] with permission from Elsevier). (c) Logarithm strain field along the tensile axis of an AZ31 magnesium alloy obtained from SEM imaging (adapted from [14]). (d) Strain field of a bimodal titanium alloy during *in-situ* test inside a SEM (adapted from [15] with permission from Elsevier). (e) Equivalent plastic strain field on the surface of a dual-phase steel studied by OM-DIC (adapted from [16] with permission from Elsevier). Strain rate field during Lüders band propagation in ultrafine-grained high-Mn austenitic steel studied by *in-situ* OM-DIC (adapted from [17]).

Originally introduced in the 1980s for optical imaging, DIC revolutionized experimental mechanics by enabling non-contact, full-field measurements of surface displacements and strains [8–10]. The method relies on tracking the relative motion of randomly distributed intensity patterns (speckles) between reference and deformed images within a selected field of view (FOV) through cross-correlation analysis. While initially developed for optical imagery, DIC has since been extended to SEM and TEM, providing access to progressively finer spatial resolutions. A comprehensive overview of the applications, advances, and challenges of DIC across OM, SEM, and TEM can be found in a recent review [11] with some examples reported in Figure 1.

Optical DIC was the first implementation of the technique and remains one of the most versatile methods for measuring surface strain across a wide range of length scales. Using visible light, it allows FOVs ranging from several millimeters (specimen-scale testing) down to tens of micrometers at high magnification, typically achieving spatial resolutions on the order of a few micrometers per pixel [18–24]. The speckle pattern used to track deformation can originate from the natural surface contrast of the specimen, such as surface roughness, or multiphase microstructures, or from artificially applied patterns created by ink spraying, particle deposition, or chemical etching [25,26]. These studies enabled full-field observation of macroscopic and mesoscopic phenomena including necking [27], Lüders band propagation [28] (Figure 1(f)), and damage nucleation [16] (Figure 1(e)), establishing optical DIC as a bridge between conventional mechanical testing and mesoscale deformation mapping [29,30].

The application of DIC to SEM images represented a major advancement, enabling submicron and nanometer-scale strain measurement by exploiting the shorter electron wavelength and raster scanning of an electron beam [31–34]. SEM-DIC offers superior spatial resolution (10–100 nm/pixel) and sensitivity to microstructural deformation features such as slip bands, grain-boundary strain localization, and microcrack initiation [15,35–40] (Figure 1 (c)). Advances in surface preparation have also been crucial, with reliable high-contrast nanometric patterns obtained via particle deposition [41–44], thin-film remodelling [35,45,46], or focused ion beam (FIB)-assisted nanopatterning [47,48]. SEM-DIC is now routinely applied to map grain-scale strain fields, identify active slip systems [49–58], and validate crystal plasticity (CP) models in metallic materials [59,60].

At the nanometer and atomic scale, DIC has recently been integrated with TEM imaging to quantify *in-situ* nanoscale strain during deformation thanks to the higher spatial resolution afforded by the greater acceleration voltage compared with SEM. In TEM-DIC, image contrast arising from the microstructure itself can be used as a natural speckle for correlation [13] (Figure 1(b)), while artificial speckles produced for instance by solid-state de-wetting of a thin film can also be used [61–64]. Compared with SEM, TEM images can be acquired at a much higher rate allowing *in-situ* monitoring with limited dwell-time. Recent developments in aberration-corrected transmission electron microscopy (Cs-TEM) have extended DIC-based strain mapping to the atomic scale by tracking the displacement of atomic columns directly in real-space images. This approach enables quantitative measurement of elastic strain and lattice rotation fields with sub-ångström precision, revealing strain accumulation and relaxation around dislocations, interfaces, and phase boundaries. Although still limited by sample thickness and FOV, Cs-TEM-based DIC provides unprecedented insight into nanoscale deformation mechanisms [12,65] (Figure 1(a)).

### 1.3. HR-DIC for sub-micron strain localization analysis

The extension of DIC from optical to electron-based imaging has enabled deformation measurements at progressively finer length scales. In this context, the so-called HR-DIC refers to DIC implementations based on high-magnification SEM imaging that achieve sub-micron or nanometer-scale spatial resolution. In addition, the HR-DIC technique is also accompanied with high resolution images with a significant number of pixels and small pixel size appropriate to evidence kinematics fields resolved at the scale of deformation of elementary deformation mechanisms, *i.e*., the nanometer scale. Importantly, ‘HR’-DIC does not rely on a fundamentally different correlation algorithm that improves sub-pixel displacement accuracy; rather, the enhanced resolution arises from the finer spatial sampling afforded by SEM imaging and speckle size. This distinction contrasts with techniques such as high-resolution EBSD (HR-EBSD), where the term ‘high resolution’ explicitly denotes improved angular sensitivity in lattice rotation and strain measurements [66]. Alternative terms such as SEM-DIC or micro-DIC (μ-DIC) are therefore utilized in the open literature [15,67–70], although these labels encompass a broad range of spatial scales and experimental configurations and may introduce additional ambiguity.

The defining feature of HR-DIC is its ability to resolve deformation at the sub-grain scale, where crystalline plasticity is inherently heterogeneous and dominated by discrete, localized events such as slip band formation, deformation twinning, and grain-boundary activity. While these mechanisms have long been studied using transmission electron microscopy, TEM observations are typically restricted to thin foils, small fields of view, and post-mortem configurations. By contrast, HR-DIC enables either *in-situ* or *ex-situ*, full-field quantification of deformation on the surface of millimeter/centimeter-size specimens under a wide range of loading and environmental conditions, thereby providing access to the spatio-temporal evolution of plasticity localization over statistically representative areas. In this respect, HR-DIC should not be regarded simply as a scaled-down variant of conventional SEM-DIC, but rather as a methodology that bridges macroscopic mechanical testing and nanoscale deformation mechanisms. In practical terms, HR-DIC typically employs subset sizes on the order of 10–50 pixels with step sizes of a few pixels, corresponding to spatial resolutions ranging from several tens of nanometers to a few hundred nanometers depending on magnification and speckle size. This enables resolution of discrete deformation features such as individual slip bands, deformation twins, and grain-boundary sliding or opening, which are generally inaccessible to conventional SEM-DIC using larger subsets and lower magnifications. In this review, the term HR-DIC therefore refers specifically to SEM-based DIC implementations designed to resolve crystal-scale deformation mechanisms through sub-micron spatial sampling combined with microstructural correlation.

The spatial resolution achievable with HR-DIC is comparable to that of EBSD, making the two techniques naturally complementary both for microstructural and deformation field characterizations. Advances in SEM instrumentation, particularly the widespread availability of field-emission gun sources, have simultaneously improved image stability for HR-DIC and pattern quality for EBSD. EBSD supplies essential microstructural features, including grain morphology, crystallographic orientation, and phase distribution, required to interpret HR-DIC strain maps, while HR-DIC provides quantitative and spatially resolved information on deformation mechanisms. Their combined use enables detailed analyses of slip-system activity, deformation twinning, and grain-boundary sliding that are not accessible using either technique separately.

In summary, HR-DIC denotes SEM-based DIC measurements performed at sub-micron length scales, typically in conjunction with microstructural characterization such as EBSD. Although the terminology is not without limitations, it has gained broad acceptance because it succinctly captures the essential capability of the technique: quantitative, full-field measurement of crystal-scale deformation in bulk materials.

### 1.4. Outline and philosophy of the present review

The present review is intended as both a conceptual framework and a practical guideline for researchers interested in using HR-DIC to study strain localization in metallic materials at the microstructure scale. Particular emphasis is placed on DIC implementations in which the subset size is sufficiently small to resolve individual deformation events, such as slip bands, deformation twins, phase transformation, or grain-boundary sliding, an approach referred here as HR-DIC. Rather than providing an exhaustive survey of all HR-DIC applications, this review focuses on the application of HR-DIC and its integration with complementary microstructural characterization methods ((HR-)EBSD, confocal microscopy), micro- and meso-scale mechanical testing, CP simulations, and recent developments in machine-learning (ML) based analysis.

HR-DIC: principle and methods introduces the experimental foundations of HR-DIC, including speckle design, SEM imaging strategies, noise sources, and correlation algorithms, with the goal of establishing best practices for acquiring robust and reproducible deformation fields. Strain localization analysis from HR-DIC results presents how HR-DIC strain maps can be quantitatively correlated with microstructure to identify active deformation mechanisms (slip, twinning, slip transfer and grain-boundary sliding) and discusses emerging data-driven and ML approaches for automated event detection, accelerated analyses, and large-scale statistical assessments. Integration with other techniques examines the integration of HR-DIC with other correlative tools, including confocal laser microscopy, HR-EBSD, *in-situ* micro-mechanical testing, and CP modeling, highlighting the added value of multi-modal and multi-scale approaches. Finally, the review concludes with a discussion of current limitations and future challenges, including the application of HR-DIC under extreme mechanical or environmental conditions and the opportunities for further methodological advances.

## 2. HR-DIC: principle and methods

The general principle of HR-DIC follows the same foundations as any DIC framework [29,30]. The method computes a two-dimensional displacement field that maps a reference (pre-deformation) image onto a current (post-deformation) image through correlation of local grayscale intensity patterns. While the overall workflow remains similar, the defining characteristic of HR-DIC is the much finer spatial resolution enabled by SEM imaging, where the subset size becomes comparable to sub-micron speckle features. It is worth mentioning that the physical kinematics related to crystal plasticity is discrete and that displacement gradients and the continuity principle inherent to conventional DIC methods are not established to address discontinuities. Therefore, DIC formulations adapted for crack detection and analysis are an alternative to better capture the discrete displacement such as pointwise DIC/subset splitting DIC [71,72], extended DIC [73,74], or Heaviside-DIC [75]. The Heaviside-DIC formulation has been specifically adapted and applied for HR-DIC to capture displacement discontinuities associated with individual slip bands [51] or deformation twinning [76]. An HR-DIC workflow typically consists of four major steps: (1) specimen preparation, microstructure characterization, and speckle-pattern application; (2) high-magnification image acquisition before and after mechanical loading; (3) computation of displacement and strain fields; and (4) post-processing, visualization, identification and quantitative analysis of the deformation mechanisms. The present section focuses on the first three steps, whereas the following section will discuss strain-field interpretation and the extraction of physically meaningful deformation metrics.

The transition from OM-DIC to SEM-DIC introduces additional experimental challenges, most notably the need to minimize imaging noise, drift, and distortion while maintaining a sufficiently high signal-to-noise ratio (SNR) at nanometer-scale pixel sizes. Ensuring stable imaging conditions, appropriate speckle morphology, and rigorous calibration is essential to fully exploit the enhanced spatial resolution that distinguishes HR-DIC from conventional SEM-DIC or OM-DIC. The following section therefore provides practical guidelines and recommended best practices for implementing HR-DIC in a reliable and reproducible manner for further standardization of the technique.

### 2.1. Speckle patterns

The spatial resolution and accuracy of DIC measurements depend critically on the quality of the applied speckle pattern. Key requirements for an effective pattern include adequate spatial randomness, suitable size and density relative to the subset dimensions, high contrast consistent with the imaging system, isotropy, and strong adhesion and stability under mechanical loading or elevated temperatures [77]. Additionally, the pattern transparency or opacity must be compatible with complementary techniques such as EBSD when correlative analyses are performed.

**Figure 2.**
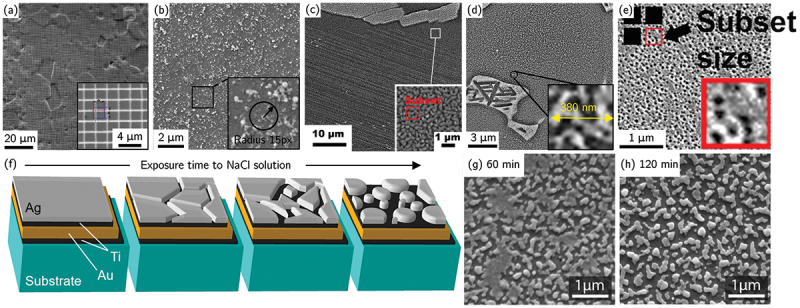
SEM images of different speckle patterns. (a) Gold microgrid deposited by micro-lithography with grid step of 2 μ m and a line thickness of 300 nm (adapted from [83] with permission from Elsevier). (b) SiO${\;}_{2}$ nano-particles (adapted from [60] with permission from Elsevier). (c) FIB speckle pattern (adapted from [48] with permission from Elsevier). (d) Gold speckle pattern produced by vapor-assisted remodeling (adapted from [97] with permission from Elsevier). (e) Etching-based speckle pattern (adapted from [38], with permission from Springer Nature). (f) Schematic of the Ag thin film reconfiguration on a multi-layer coated substrate when exposed to NaCl solution and (g–h) corresponding pattern after (g) 60 min and (h) 120 min immersion in a 1 wt.undefined NaCl solution (adapted from [45], with permission from Springer Nature).

A wide range of patterning methods have been developed and reviewed [41,77], including paint or ink deposition [26,78,79], microgrids [31,44,80–84], chemical etching [38,85], particle deposition [59,60,86–90], micro-/nano-printing [91,92], focused ion beam (FIB) speckle milling [48], focused ion or electron-beam induced (Pt) deposition (FIBID & FEBID) [93,94], direct sputter deposition [42–44] and thin-film remodelling [35,45,95,96] (Figure 2). A sequential combination of different deposition techniques, *e.g*. direct sputter deposition and electron lithography-based microgrids, can allow for multiscale characterization of the deformation of materials [44].

The thin-film remodeling method is widely adopted in the literature for HR-DIC and relies on dewetting of a uniformly deposited metallic nanolayer exposed to condensable vapors, producing a network of isolated nanoparticles whose morphology depends on the film thickness, the substrate surface condition, and the exposure time [47,98]. While water vapor was originally used, a styrene-assisted gold remodelling device was also developed and applied to materials susceptible to corrosion under hot water vapor such as Mg alloys [14,99,100]. In contrast, direct deposition of metallic or colloidal nanoparticles often produces non-uniform or clustered patterns, restricting DIC measurements to regions with well-dispersed particles and limiting quantitative strain analysis over larger FOVs [88]. Although highly tunable, thin-film remodelling can be sensitive to substrate condition. To overcome this limitation, multilayer deposition strategies have recently been developed to ensure substrate-independent pattern formation [45] (Figure 2(f–h)). After sequential deposition (*e.g*. Ti/Au/Ti/Ag stacks), the sample is immersed in a 1 wt.% NaCl aqueous solution, where the Ag layer thickness and immersion duration govern the morphology of the resulting speckle pattern. The obtained pattern was found to be stable under cryogenic temperature when the sample was exposed to liquid nitrogen and helium [76]. To avoid corrosion issues on reactive substrates such as Cu and its alloys, a recent modification replaces the NaCl – water system with NaBr dissolved in isopropanol, producing high-quality speckle patterns suitable for both HR-DIC and HR-EBSD analyses [46]. Alternatively, to avoid the need for remodelling, a direct one-step deposition of a low melting temperature solder alloy (*e.g*. InSn, Sn, In) were proposed, naturally forming a speckle pattern with feature sizes that depend on the applied sputtering conditions [42–44,62,101]. However, the resulting patterns are typically too dense to also enable EBSD.

For HR-DIC, achieving submicron subset sizes is essential to resolve individual slip traces or micro-bands. The reported subset sizes for the different pattern conditions are summarized in Table 1. Sub-micron subset size has been reported for etching, particle deposition, direct sputter deposition, electron-beam deposition, and thin-film remodelling, which have become the most common techniques in HR-DIC applications. Depending on the speckle type, either secondary electron (SE) or backscattered electron (BSE) imaging modes are selected to optimize contrast and noise performance.

**Table 1.** Summary of pattern techniques and their achievable subset size.

Patterning method		Substrate	Imaging mode	Subset size	Ref.
Paint/ink		Al-Mg	OM	268x268 μm${\;}^{2}$	[78]
		Al-Mg	SEM-SE	70x70 μm${\;}^{2}$	[79]
		Ti	OM	6x6 μm${\;}^{2}$	[26]
Microgrid		LIGA	SEM-BSE	21x21 μm${\;}^{2}$	[80]
		LIGA	SEM-BSE	10x10 μm${\;}^{2}$	[81]
		IF-Steel	SEM-SE	5x5 μm${\;}^{2}$	[82]
		Zr/Ti	OM/SEM-SE	2x2 μm${\;}^{2}$	[83]
Etching		Cu	SEM-SE	1.26x1.26 μm${\;}^{2}$	[85]
		Ni	SEM-SE	400x400 nm	[38]
FEBID	Pt	Ni	SEM-SE	7x7 μm${\;}^{2}$	[86]
	Pt	TiAl	SEM-SE	108x108 nm${\;}^{2}$	[94]
Particle deposition	Al${\;}_{2}$O${\;}_{3}$	Mg	SEM-SE	12.45x12.45 μm${\;}^{2}$	[59]
	SiO${\;}_{2}$	Steel	SEM-SE	1.14x1.14 μm${\;}^{2}$	[90]
	SiO${\;}_{2}$	Steel	SEM-SE	360x360 nm${\;}^{2}$	[60]
	Au	Mg	SEM-SE	1.85x1.85 μm${\;}^{2}$	[68]
Sputter deposition	InSn	Fe	SEM-SE	336x336 nm${\;}^{2}$	[42]
	InSn	Steel	SEM-SE	105x105 nm${\;}^{2}$	[102]
	InSn	Zn	SEM-SE	294x294 nm${\;}^{2}$	[103]
	In	Al	SEM-SE	165x165 nm${\;}^{2}$	[62]
	In	Pd ${\;}_{71}$Si ${\;}_{29}$	SEM-SE	195x195 nm${\;}^{2}$	[104]
FIB speckling		Mg	SEM-SE	1.12x1.12 μm${\;}^{2}$	[48]
Thin film remodelling	Ti/Ag	CuCrZr	SEM-BSE	272x272 nm${\;}^{2}$	[46]
	Ti/Au/Ti/Ag	Ti	SEM-SE	240x240 nm${\;}^{2}$	[45]
	Au	Steel	SEM-BSE	216x216 nm${\;}^{2}$	[35]
	Au	Steel	SEM-BSE	81x81 nm${\;}^{2}$	[96]

**Figure 3.**
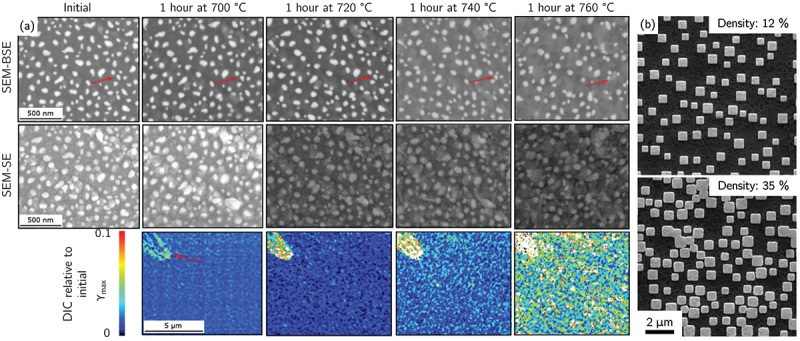
(a) Backscatter and secondary electron images of an Au speckle pattern on a TiAl substrate following successive temperature holds and corresponding maximum shear strain map estimated by DIC relative to the initial state (adapted from [47]). (b) SEM images of hafnium oxide speckles deposited by electron-beam lithography (adapted from [105], with permission from Springer Nature).

The thermal stability of speckle patterns at elevated temperature is a critical consideration for HR-DIC measurements aimed at probing temperature-dependent mechanical behavior, such as creep or thermally activated deformation mechanisms. Thin film remodelling of Au speckles has been shown to retain sufficient contrast and morphological stability for approximately one hour at temperatures up to 720° C on TiAl substrates [47] (Figure 3). Under these conditions, BSE imaging provides superior pattern contrast compared with SE imaging, as the latter becomes more sensitive to oxide formation between speckles. However, at higher temperatures or longer exposure times, the strain fields exhibit a significant increase in noise, restricting the applicability of Au-based patterns to short-duration monotonic tests. Microstructure-based speckle pattern (etching plus slight pre-oxidation) for two/three-phase Ni-based superalloys have also demonstrated potential use for *ex-situ* HR-DIC experimental [106–109]. Temperature limitation at 650° C comes also from the speckle evolution due to high temperature oxidation in air [106] or under vacuum [107–109], especially for materials exhibiting a cationic oxidation behavior. For more demanding thermal environments, alternative patterning strategies have been developed. Hafnium oxide speckles deposited on nickel-base superalloys using electron-beam lithography exhibit excellent contrast, oxidation resistance, and long-term thermal stability at 700° C. These patterns consist of randomly distributed diamond-shaped features with edge lengths of 400–700 nm or micro-grids, providing a robust and thermally stable speckle morphology [105,110–114] (Figure 3(b)). Temperatures up to 1000 C° were also achieved using very fast thermomechanical Gleeble testing to limit oxidation processes [115]. Finally, vapor-assisted remodelling of Au nanolayer was also found to be resilient to proton irradiation for doses between 0.1 and 2 dpa up to a moderate irradiation temperature of 650 ${\;}^{\circ }$ C [116,117].

To evaluate the speckle-pattern quality and determine the optimal subset size, several local indicators have been proposed based on gray-level variability within subsets, as DIC noise is strongly influenced by local intensity fluctuations. Common metrics include the sum of squared intensity gradients [118], the mean intensity gradient [119], the mean second derivative of intensity [120], the mean subset fluctuation [121], the subset-size-dependent mean subset fluctuation [47], and the entropic variability [122]. Careful adjustment of SEM imaging parameters, particularly brightness and contrast, can maximize the effective dynamic range of the grayscale histogram while avoiding saturation at either end, thereby enhancing these indicators and improving correlation quality.

### 2.2. Image acquisition and noise sources

**Figure 4.**
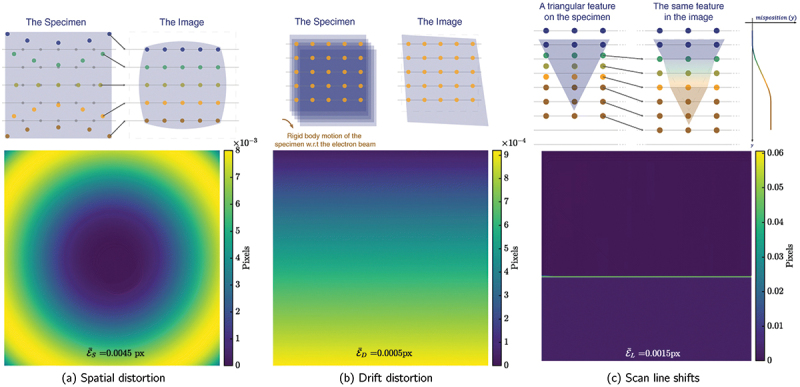
Schematic illustrations of (a) spatial distortion, (b) drift distortion and (c) scan line shift and examples of corresponding displacement amplitude fields. The gray horizontal lines indicate the ideal scan lines, *i.e*., where the scanning should have happened in case of no beam positioning error. Colored dots represent where scanning actually occurred (adapted from [132]).

SEM enables imaging at far higher spatial resolution than optical systems owing to the much shorter wavelength of accelerated electrons compared with visible light. However, the accuracy and precision of SEM-based DIC measurements are highly sensitive to the SNR and spatial repeatability of the acquired images. Numerous instrumental and environmental factors can introduce image distortions and noise, which in turn cause virtual pixel displacements and generate artificial strain fields during DIC analysis [33,34,36,38,68,123–132]. The sources of image distortion in SEM can be broadly categorized into four main types: (1) spatial distortion, (2) drift distortion, (3) scan-line shift, and (4) scan encoding [132] (Figure 4).

Spatial distortion is a systematic, time-independent aberration analogous to optical lens distortion, arising from inhomogeneities in the electromagnetic field of the objective lens. These field variations cause nonuniform deflection of the electron beam, resulting in positional errors across the image field (Figure 4(a)). Spatial distortion typically manifests as a smooth, monotonic warping of the image geometry and is most pronounced at low magnifications (below 400 × magnification/FOV of approx. 500 µm), where the beam scans over a large area. Because this distortion is tied to the system, it remains consistent across all images as long as the SEM parameters (*e.g*., working distance, accelerating voltage, etc) remain unchanged [34,36].

Drift distortion, by contrast, is a time-dependent and non-random artifact caused by undesired deflection of the electron beam or unwanted movement of the samples during image acquisition. The main contributors include specimen charging, thermal expansion or contraction, creep in sample when at load, movement of the loading stage or sample holder, and vibrations. These effects collectively lead to a progressive shift of the FOV during scanning, which translates into non-uniform stretching, shrinking, or shearing of the final image (Figure 4(b)). Unlike spatial distortion, drift distortion varies from one image to another and becomes increasingly severe at high magnifications due to the longer acquisition times and smaller scanned areas [34].

Finally, scan-line shifts represent random, time-independent events typically caused by transient beam positioning errors. These are believed to result from the sudden discharge of contaminant particles within the electron column [130,132]. The resulting error produces abrupt discontinuities in the raster scan, leading to single-pixel wide displacement bands or step-like features in the image (Figure 4(c)). Such events can generate large apparent local strain artifacts that are easily misinterpreted in DIC analyses if not detected and corrected. These artifacts are more pronounced on fields of the displacement component normal to the scanning direction (*e.g*., along Y if the scanning direction is along X), leading in a certain anisotropy of this artifact. An external control of the physical beam scanning was found to clearly reduce line shift artifacts [133].

Spatial and drift distortions can be reduced by taking high magnification images using a large spot size, a long dwell time and a low acceleration voltage [38]. A magnetic immersion lens can also be used to decrease the spot size and improve the resolution at lower magnification [134]. Several additional strategies have been developed to mitigate SEM imaging artifacts in DIC analysis [34,130,132,135]. Early correction schemes combined sequential image correlation to quantify drift distortion with calibration procedures to compensate for spatial distortion [34]. Later developments introduced reference grids and optimized imaging parameters to minimize noise, drift, and magnification errors during long-duration or *ex-situ* tests [131]. Recent approaches have formulated frameworks in which spatial, drift, and scan-line distortions are simultaneously identified and corrected through hierarchical mapping functions within the DIC optimization process, achieving sub-pixel correction accuracy [130,132]. Alternatively, a more practical but less precise correction method involves acquiring two images at each deformation step with orthogonal scanning directions (by applying a 90 ${\;}^{\circ }$ scan rotation for the second scan), which are then ‘combined’ into a distortion-free scan by applying global DIC on the individual scan lines [69]. Non-affine distortion can also be corrected using overlapped regions when mosaic of images are performed to cover a large field of view with a fine spatial resolution [136].

A less frequently discussed but equally important source of uncertainty arises from the SEM magnification itself. Manufacturers typically specify a magnification accuracy on the order of a few percent, and even nominally identical imaging conditions can yield small scale-factor differences between sessions (homothetic distortion). Such variations can translate directly into fictitious strains that may overshadow the true material response in low-strain experiments [131]. Consistency checks, such as acquiring reference images (*e.g*., calibration grids) at each session and correcting for offsets or magnification drift, help suppress this artificial strain. Moreover, because SEM magnification often exhibits hysteresis, beginning each imaging sequence from a high magnification and stepping down to the working magnification can improve repeatability.

While these distortion mechanisms can significantly influence the quality and noise level of HR-DIC strain fields, particularly in the elastic regime where the strain level is very low [38], it is important to note that most HR-DIC studies do not apply explicit distortion-correction algorithms. Instead, DIC is often performed directly on the acquired SEM images [14,35,60,90,96,97,137–139]. This practice is partly enabled by the substantial improvements in modern SEM instrumentation, including enhanced beam stability, field-emission sources, and optimized electronic control systems, but also by systematic investigations that clarified how acquisition and algorithm parameters affect noise levels [38,105]. In practice, factors such as working distance, beam current and accelerating voltage, stigmatism correction, charging-induced drift, magnification, dwell time, spot size, and image resolution typically exert a far greater influence on DIC noise than the intrinsic distortion sources of the SEM itself. Therefore, maintaining these parameters consistent and well-optimized throughout acquisition is essential for reliable HR-DIC measurements.

### 2.3. DIC algorithms

**Figure 5.**
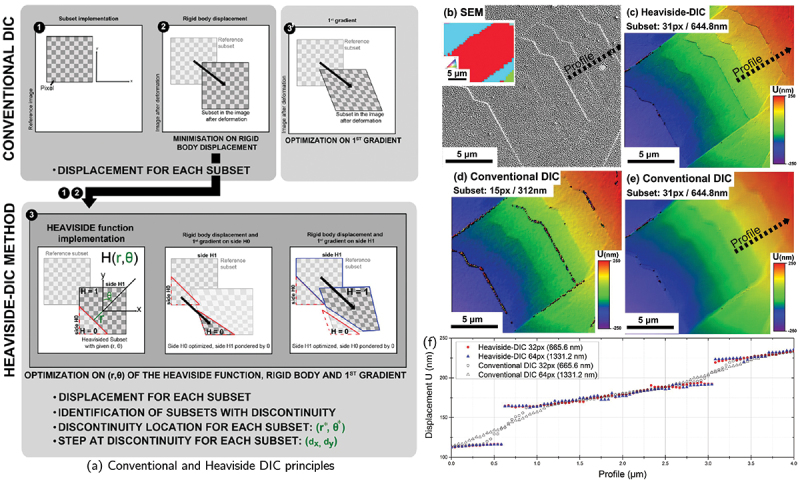
(a) Schematic diagram of the conventional and Heaviside DIC methods. (b) SEM image after 1 % macroscopic deformation of a nickel-based superalloy. (c–e) Corresponding displacement amplitudes calculated by (c) Heaviside-DIC with a subset of 31 pixels and (d–e) conventional DIC calculated with a subset of 15 and 31 pixels, respectively. (f) Displacement profile along the path highlighted in (b–e) (adapted from [51], with permission from Elsevier).

DIC quantifies in-plane displacement fields by tracking the motion of intensity patterns between a reference (undeformed) image and a current (deformed) image. The general principles of DIC have been thoroughly reviewed elsewhere [29,30]. Here, we briefly summarize the formulation of the conventional subset-based DIC approach and introduce the Heaviside-DIC formulation, which incorporates displacement discontinuities to capture localized shear bands or slip lines that cannot be accurately described by standard continuous mappings [48,51,53,140–144]. The principle of the two DIC approaches is depicted in Figure 5(a) [51].

#### 2.3.1. Conventional DIC formulation

In conventional DIC, a small interrogation window (subset) of size 2R+1 and centered at $X_{0}=(x_{0},y_{0})$ in the reference image, contains pixels at positions $X=(x_{i},y_{j})$, (i,j)∈[−R:R]×[−R:R]. During deformation, the corresponding pixel in the current image is located at $X^{*}$. The mapping from X to $X^{*}$ is typically approximated by a first-order displacement field:

(1) $$X^{*}=X+U_{r}+\nabla U_{s}.(X-X_{0})$$

where $U_{r}$ is a rigid body translation of the subset center and $\nabla U_{s}$ is the local deformation gradient describing normal and shear deformation within the subset. Higher order shape functions (e.g. quadratic) can also be employed. The unknown deformation parameters are obtained by minimizing a similarity metric (*e.g*., normalized cross-correlation or sum of squared differences) between the reference and warped subsets. This approach assumes a continuous displacement field inside each subset and therefore cannot represent sharp displacement jumps when a displacement discontinuity belongs to consecutive subsets. Techniques such as subset truncation [71,72], or eXtended DIC (X-DIC) [73,74], inspired by the eXtended finite element method (X-FEM) allow an improved solution for the displacement field across discontinuities but is mostly applied for calculating displacement fields around cracks [21].

#### 2.3.2. Heaviside-DIC formulation

To account for localized, discontinuous displacement fields, such as those produced by slip bands, the conventional DIC mapping can be enriched with an additional discontinuity term weighted by a Heaviside step function [51,75]:

(2) $$X^{*}=X+U_{r}+\nabla U_{s}.(X-X_{0})+U_{h}.H(X-X_{0})$$

where $U_{h}$ is the displacement jump amplitude across the discontinuity and H(⋅) is the Heaviside function identifying the two sides of the discontinuity. If the subset size is small compared with the discontinuity, the segment of the discontinuity crossing the subset can be assumed to be linear. A linear discontinuity is defined by two parameters: $r^{*}$ the perpendicular distance from the subset center to the discontinuity line and $\theta^{*}$ the orientation of the normal vector $n^{*}=(\cos\theta^{*},\sin\theta^{*})$. In Cartesian coordinates, the discontinuity line is given by:

(3) $$L(r^{*},\theta^{*})=\left\{X\in R^{2}\;:\;(X-X_{0})\cdot n^{*}=r^{*}\right\}$$

The signed distance from a point to the discontinuity is:

(4) $$d(X-X_{0})=(X-X_{0})\cdot n^{*}-r^{*}$$

and the associated Heaviside function is:

(5) $$H(X-X_{0})=\left\{\begin{array}{cc} 0, & d(X-X_{0})\leq 0,\\ 1, & d(X-X_{0})>0. \end{array}\right.$$

This partitions the subset into two domains, H0 (H=0) and H1 (H=1), across which a finite displacement jump $U_{h}$ is permitted. Conventional DIC is then independently and simultaneously performed to both domains, and the correlation procedure is repeated for multiple combinations of ($r^{*}$,$\theta^{*}$). In the absence of an actual discontinuity within the subset, the optimized displacement field converges to the same result as conventional DIC. The enriched formulation introduces additional unknowns, the displacement jump components and the geometric parameters ($r^{*}$,$\theta^{*}$), as well as separate rigid-body and deformation-gradient terms for each side of the subset. Consequently, the computational cost of Heaviside-DIC is typically about four times higher than that of conventional DIC and the minimum subset size is also larger [51]. Figure 5(b–f) compares displacement fields obtained using conventional and Heaviside DIC. In regions free of discontinuities, the two methods produce nearly identical results (Figures 5 (5–d)). However, across an actual slip band, conventional DIC is not capable to converge for subsets at the discontinuity (small subset size) or yields an artificially smooth displacement transition whose width depends on the subset size (large subset size), whereas Heaviside-DIC correctly identifies a sharp displacement jump independent of the subset size. Moreover, the apparent intensity of plastic deformation at slip bands in conventional DIC is highly sensitive to correlation parameters such as the measurement step and subset size, while Heaviside-DIC provides a more physically meaningful measure of displacement discontinuity.

#### 2.3.3. Adaptive particle image velocimetry (PIV) algorithms

Since several of the patterning methods produce high contrast speckle patterns, they can be successfully analyzed using algorithms designed for particle image velocimetry. In most high magnification HR-DIC experiments, the speckles themselves are not deformed and therefore the images deform like those in a PIV experiment. These algorithms are designed to track the movement of particles and do not assume continuous deformation, and therefore have the advantage that they can capture jumps in displacement better than conventional DIC [134], whilst still measuring diffuse strain. In PIV analysis, it is common to use iterative and adaptive algorithms that can iteratively deform and decrease the sub-region size during the analysis, improving its ability to measure deformation when the local deformation is complex. This type of algorithm is available in earlier versions of the commercially available DaVis (LaVision) software but also through open source packages like OpenPIV [145] and others, which are GPU accelerated, and can be easily integrated into an HR-DIC workflow.

#### 2.3.4. Correlation criterion, algorithms and sub-pixel accuracy

The degree of similarity or difference between the reference and deformed subsets can be quantified by various cross-correlation (CC) or sum of squared differences (SSD) criteria based on the gray-level pattern within each subset [29]. CC-based algorithm identifies the displacement producing the highest correlation coefficient between subsets, typically accelerated using Fast Fourier Transform (FFT) implementations to enable efficient pixel-wise searching, while it is the opposite for SSD-based algorithms. Because integer-pixel matching limits accuracy, this is followed by sub-pixel refinement, commonly achieved through least-squares matching (LSM), which minimizes the grey-level residuals while accounting for affine transformations within the subset and thus provides robust sub-pixel displacement estimation. The main classes of correlation criteria, sub-pixel algorithms, including coarse – fine search, peak-finding via polynomial or Fourier surface fitting, and iterative spatial-domain cross-correlation has been summarized in dedicated review [29].

**Figure 6.**
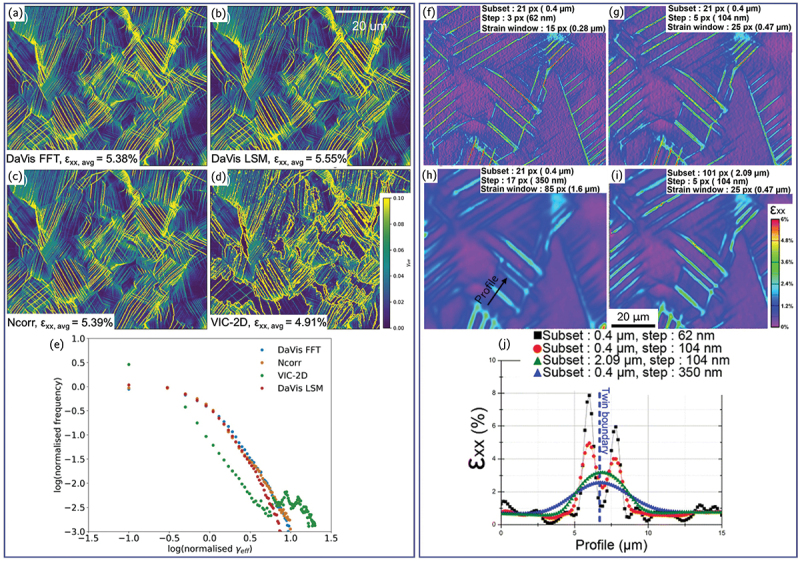
(a–d) Effective in-plane shear strain maps for a Ti-6Al–4 V alloy deformed at about 5.0 % strain calculated with different DIC software and (e) corresponding normalized frequency distributions (adapted from [134]). (f–i) $\varepsilon_{\mathrm{xx}}$ strain field maps of a nickel-based superalloy deformed at 0.98 % tensile strain calculated with different subset size, step and strain window and (j) $\varepsilon_{\mathrm{xx}}$ strain along the profile path depicted in (h) (adapted from [38], with permission from Springer Nature).

A variety of commercial and open-source DIC packages are commonly used, such as VIC-2D (Correlated Solutions, US) [68,146], DaVis (LaVision, Germany) [14,47,147], MatchID [69,102], Ncorr [148], py2DIC [149], each implementing different correlation, interpolation, and optimization strategies, which can lead to non-negligible discrepancies, especially in high-resolution applications. A recent HR-DIC benchmark study comparing DaVis FFT, DaVis LSM, Ncorr, and VIC-2D showed that although these methods agree under relatively homogeneous deformation, substantial differences appear when analyzing sharp strain gradients such as slip bands but also homogeneous deformation between slip bands [134]. For example, VIC-2D exhibited difficulty correlating high-strain features unless the interrogation window and step size were finely tuned, whereas DaVis and Ncorr produced more reliable displacement fields under identical conditions (Figures 6(a)). These discrepancies emphasize that HR-DIC results are software-sensitive, and that optimal correlation parameters must be carefully selected and validated to avoid artifacts such as smoothed-out slip bands or spurious displacement tails reported in some implementations.

#### 2.3.5. Machine-learning-based DIC approaches

Recent advances in deep learning for computer vision have shifted DIC from an optimization-based technique into a fast, data-driven framework capable of estimating displacement fields with dramatically reduced computational time. Early architectures, such as StrainNet [150] and DIC-Net [151] based on FlowNet-type convolutional neural networks (CNNs) [152], demonstrated that CNN can approximate optical-flow mappings between reference and deformed images, enabling near-real-time DIC inference once trained. However, purely data-driven models often suffer from limited generalization as their accuracy degrades when speckle characteristics deviate from the training set, when noise levels increase, or when natural textures replace artificial high-contrast patterns. To address these limitations, several hybrids physics-informed strategies have been proposed [153–157] and demonstrated higher robustness compared with conventional DIC algorithms, especially for highly localized strain and deformed pattern. So far, these approaches have mostly been applied to optical DIC dataset and remain to be used in HR-DIC/SEM-DIC studies.

### 2.4. Computation of strain fields

Once the in-plane displacement fields u=(u,v) are obtained from the correlation procedure, they can be used to compute the corresponding strain fields. Under a finite-strain description, the in-plane components of the Green – Lagrange strain tensor are expressed as:

(6) $$E=\frac{1}{2}\left(\nabla u+{\left(\nabla u\right)}^{T}+{\left(\nabla u\right)}^{T}\nabla u\right),$$

In many DIC applications, particularly those involving small deformations, the quadratic terms are neglected and the small-strain approximation is adopted. Although the displacement gradients can be directly obtained from the correlation solution, these gradients are generally noisy and are generally considered unreliable for strain estimation [29], although they can be useful when using a larger subset size [69]. Instead, strain fields are typically computed by fitting the displacement field within a small interpolation zone, often termed the strain calculation window, using a local linear approximation:

(7) $$u(X)\approx u(X_{0})+\nabla u.(X-X_{0})$$

where the coefficients are obtained by a least-squares regression over the chosen window. Importantly, the strain window size is independent of the subset size used during the correlation step, but it does change with the subset spacing (or step size).

Assuming small deformation, a number of scalar quantities are commonly derived from the in-plane strain tensor and used for visualization and quantitative analysis of DIC results. A first, very intuitive measure is the maximum in-plane shear strain, defined as

(8) $$\gamma_{\max}=\sqrt{{\left(\frac{\varepsilon_{\mathrm{xx}}-\varepsilon_{\mathrm{yy}}}{2}\right)}^{2}+\varepsilon_{xy}^{2}}.$$

A second, widely used measure is the equivalent (von Mises) plastic strain, which provides a scalar measure of the accumulated deviatoric strain:

(9) $$\varepsilon_{\mathrm{eq}}=\frac{2}{3\sqrt{2}}\sqrt{{\left(\varepsilon_{\mathrm{xx}}-\varepsilon_{\mathrm{yy}}\right)}^{2}+{\left(\varepsilon_{\mathrm{yy}}-\varepsilon_{\mathrm{zz}}\right)}^{2}\;+{\left(\varepsilon_{\mathrm{zz}}-\varepsilon_{\mathrm{xx}}\right)}^{2}+6\left(\varepsilon_{yz}^{2}+\varepsilon_{zx}^{2}+\varepsilon_{xy}^{2}\right)}.$$

In DIC applications, where only the in-plane components are directly measured, a plane-stress assumption can be adopted, so that

(10) $$\varepsilon_{\mathrm{zz}}=-\frac{\nu }{1-\nu },(\varepsilon_{\mathrm{xx}}+\varepsilon_{\mathrm{yy}}),$$

with ν the Poisson’s ratio and $\varepsilon_{\mathrm{yz}}=\varepsilon_{\mathrm{zx}}=0$. Under these assumptions, $\varepsilon_{\mathrm{eq}}$ can be evaluated from the in-plane strain components. Both $\gamma_{\max}$ and $\varepsilon_{\mathrm{eq}}$ highlights regions of intense deformation and are particularly useful for revealing slip bands, shear bands, and grain-boundary sliding.

In addition to strain-based scalars, material rotation is often extracted from the displacement field. For small deformation, the out-of-plane component of the rotation vector is given by

(11) $$\omega_{z}=\frac{1}{2}\left(\frac{\mathrm{\partial v}}{\mathrm{\partial x}}-\frac{\mathrm{\partial u}}{\mathrm{\partial y}}\right)$$

Maps of $\omega_{z}$ provide insight into lattice or grain-block rotation and are particularly informative near grain boundaries, twin boundaries, or within shear bands where significant lattice reorientation occurs. This rotation can be seen to agree well with EBSD misorientation, when transformed into a reference frame aligned with the slip bands [137]. Rotation fields are also implemented in the Heaviside-DIC algorithm to measure in-plane rotation between slip bands for direct comparison with EBSD characterizations [158].

For HR-DIC, both a small subset size and a small strain window are necessary to resolve individual deformation bands with high fidelity [38,159]. Figure 6 (f) illustrates the strong influence of subset size, subset spacing, and strain window on the reconstructed strain fields [38]. Note that the subset spacing and strain window are tightly related. For example, 5 ‘displacement datapoints’ in Figure 6 (f) are used to compute the strain, but the subset spacing is different, involving changes in strain window. Two distinct and closely spaced slip bands lie on either side of a twin boundary in a deformed nickel-based superalloy. Enlarging either the subset size or the strain window causes the two bands to merge into an apparently single feature centered on the twin boundary. Improper parameter selection can therefore shift the apparent position of strain localization and lead to incorrect mechanistic interpretations. However, the use of very fine subsets and strain windows, while necessary for spatial resolution, may introduce significant high-frequency noise. Several filtering strategies are commonly employed to mitigate this effect, including applying a Gaussian filter to the displacement fields (acquired using very small subset spacing) prior to differentiation [69,160], or applying low-pass FFT filtering directly to the strain fields [38]. Total variation filtering can also be used to remove high frequency noise but preserve sharp discontinuity in kinematics fields due to slip bands [161]. Therefore, a balance must be achieved between spatial accuracy and measurement noise, with the optimal subset size depending on both the speckle pattern characteristics and the spatial frequency of the underlying deformation. Moreover, because the amplitude of the measured strain fields is directly dependent on these correlation parameters in the case of conventional DIC, meaningful comparisons of strain distributions, whether across different applied strain levels, microstructures, or materials, require that the same subset size, spacing, and strain window be consistently maintained.

## 3. Strain localization analysis from HR-DIC results

### 3.1. Coupling HR-DIC with EBSD data

Although HR-DIC provides a quantitative map of strain localization, its full interpretive power emerges when strain fields are coupled with microstructural information obtained from EBSD. By correlating DIC and EBSD, one can directly link slip activity, strain concentration, or deformation incompatibilities to crystallographic orientation, grain-boundary character, phase distribution, or local heterogeneities. Achieving this correlation, however, requires accurate spatial alignment between the strain map and the EBSD scan. EBSD maps, especially those acquired over large FOV at low magnification, are susceptible to geometric distortions arising from sample tilt, beam scanning nonlinearities, and drift during long acquisitions [68,162–165]. Tilting the specimen by approximately 70° for EBSD projects an initially square region into a trapezoid, introducing an affine distortion that can generally be corrected using a first-order affine transformation. Spatial distortion as described in the previous section can be corrected using a second-order polynomial function that accounts for the nonlinearity of the beam path. Unlike tilt and spatial distortions, temporal drift due to specimen charging, beam instability, or stage drift has no straightforward analytical correction and must be estimated empirically.

**Figure 7.**
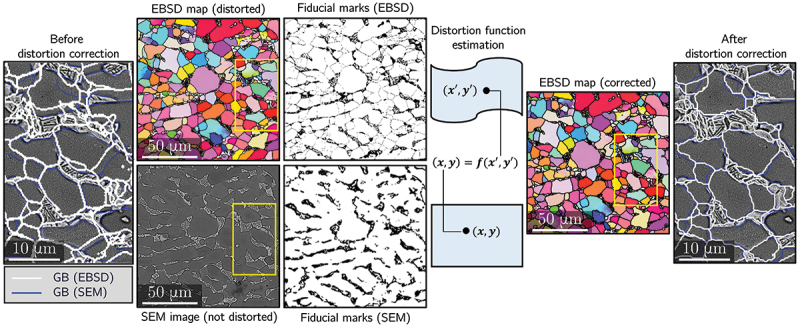
General overview of a procedure for correction of the spatial distortion in EBSD maps based on the creation of a distortion function mapping pixels from the EBSD map to its corresponding position in a reference image (adapted from [97], with permission from Elsevier).

To register EBSD data to the strain map, most approaches determine a distortion field that maps the EBSD coordinates onto a reference SEM image acquired at 0 ${\;}^{\circ }$ tilt. This is typically achieved by matching homologous microstructural features, such as grain boundaries, triple junctions, or phase interfaces, and fitting a distortion function to their spatial offsets (Figure 7). Third-order polynomial warping functions are widely used [58,69,166], although more advanced feature-matching methods (*e.g*., evolutionary optimization [53,164], spline-based interpolations [167], and hybrid polynomial – spline corrections [165]) have also been adopted. Recent work has demonstrated that displacement fields derived directly from DIC can serve as correction fields for EBSD scans, offering a unified and physically grounded approach to map registration [97,160].

In addition to geometric distortion correction, practical constraints related to the speckle pattern itself must also be considered when coupling HR-DIC with EBSD. In many experiments, the speckle layer used for DIC partially obscures Kikuchi patterns or add additional diffraction features, preventing high-quality EBSD acquisition during deformation. One approach is to perform EBSD before applying the speckle pattern [69,102], after which data is aligned as described above. Another strategy is to acquire EBSD only after mechanical testing, once slip traces and strain patterns have formed [54,87,168]. In this post-mortem workflow, the speckle pattern is removed, either mechanically (*e.g*., gentle polishing, broad ion beam cleaning) or chemically (*e.g*., selective etching of Au, Ag, Pt coatings), before EBSD mapping. This approach avoids the need for transparent speckle patterns but restricts correlation to a single deformation state and requires careful surface preparation to avoid altering slip markings or to much material removal for alignement [169].

An alternative to post-mortem pattern removal is to employ speckle patterns that are intrinsically compatible with EBSD. For example, amorphous SiO ${\;}_{2}$ (or other) nanoparticle coatings produce high-contrast HR-DIC speckle patterns while remaining effectively transparent to the electron beam, enabling EBSD acquisition without removing the pattern [88,89,170,171]. Similarly, when Au-based speckle layers are deposited with sufficiently small thickness, typically on the order of a few nanometers, the Kikuchi patterns remain of adequate quality for reliable indexing [96]. These speckle patterning strategies allow sequential HR-DIC and EBSD mapping during an experiment, eliminating the need for post-test pattern removal and preserving slip traces that might otherwise be damaged.

### 3.2. Strain localization characterization

#### 3.2.1. Crystallographic slip

In most metallic materials, plastic deformation is dominated by crystallographic slip, resulting from the collective motion of dislocations along well-defined crystallographic planes and directions. Identifying the active slip systems during deformation at the grain level is therefore essential for understanding the operative mechanisms that control plasticity.

Traditionally, slip trace analysis has been the most common technique for such identification. By analyzing the orientation of slip lines visible on the specimen surface, one can infer the active slip plane, especially in cases of planar slip. However, determining the corresponding slip direction is more challenging, as it often relies on additional assumptions, such as the selection of the system with the highest Schmid factor (SF) or based on the relative magnitudes of the critical resolved shear stresses (CRSS) among systems sharing the same slip plane [172]. These assumptions introduce uncertainties, particularly in materials with multiple slip families or near-equivalent systems, such as face-centered cubic (FCC) or hexagonal close-packed (HCP) metals.

**Figure 8.**
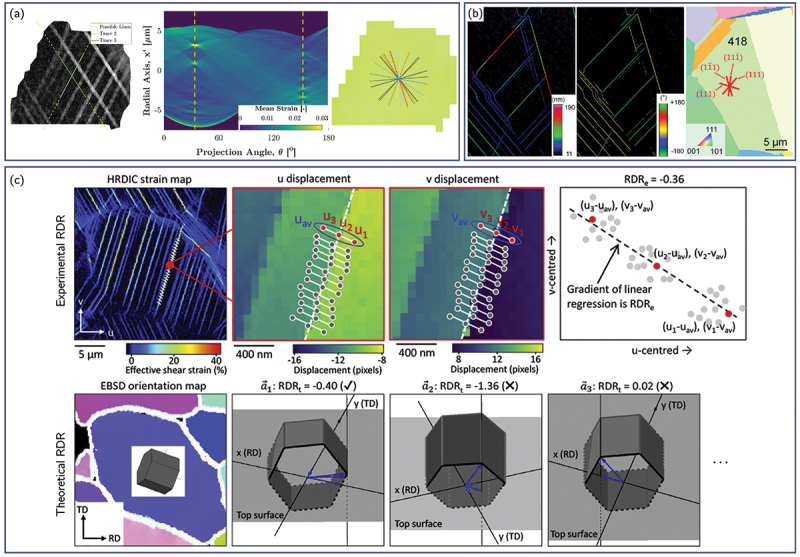
(a) Automated identification of slip plane by Radon-transform slip trace analysis (adapted from [97], with permission from Elsevier). (b) Slip system identification based on probabilistic Hough transform of Heaviside-DIC displacement map (adapted from [53], with permission from Elsevier). (C) Principle of the identification of the slip direction based on the comparison of the experimental and theoretical RDR (adapted from [52]).

To overcome these limitations, recent developments have leveraged the full-field displacement and strain data obtainable from HR-DIC to identify active slip systems more reliably. Assuming planar slip, individual slip bands or traces within a grain can be detected directly from the DIC strain maps using automated feature-extraction techniques, such as physics-based projection techniques based on EBSD data [173], the Hough transform [53,142,174] or the Radon transform [58,97,138,139,175] (Figures 8(a)). These detected slip traces can then be compared to the theoretical projections of the crystallographic slip planes onto the 2D specimen surface, allowing a direct correlation between experimental slip features and the underlying crystal geometry. To ensure accurate matching between experimental and theoretical traces, it is often necessary to account for lattice rotation and grain-shape evolution during deformation. Accordingly, the experimental strain or displacement fields can be represented in the undeformed configuration, thereby minimizing alignment errors.

To further clarify the slip direction, quantitative criteria based on the HR-DIC data can be used. Under the assumption of single slip, the Relative Displacement Ratio (RDR) between the x- and y-components of the displacement field across a slip line can be experimentally determined as [49]:

(12) $$\mathrm{RD}R^{\exp}=\frac{\Delta u_{x}}{\Delta u_{y}}$$

where $u_{x}$ and $u_{y}$ represent the displacement components along the in-plane directions. This experimental ratio can then be compared with the theoretical RDR, defined for a slip system α characterized by a slip plane of unit normal $n^{\alpha }$ and slip direction $s^{\alpha }$:

(13) $$\mathrm{RD}R^{\alpha }=\frac{\Delta s_{x}^{\alpha }}{\Delta s_{y}^{\alpha }}$$

where $s_{x}^{\alpha }$ and $s_{y}^{\alpha }$ are the in-plane components of the slip direction vector. Because coplanar slip systems with different (in-plane projections of) Burgers vectors exhibit distinct theoretical $\mathrm{RD}R^{\alpha }$ values, comparing $\mathrm{RD}R^{\exp}$ and $\mathrm{RD}R^{\alpha }$ enables reliable identification of the activated slip system [49,52,58,174] (Figure 8(c)). Other, more advanced methodologies have also been developed to improve slip system identification by exploiting the full-field displacement data obtained from DIC. For example, as Heaviside-DIC approaches explicitly capture the discontinuous displacement fields associated with slip events, it can directly provide the discrete in-plane slip vectors corresponding to each detected slip trace [51,53,54]. Alternatively, optimization-based techniques have been introduced in which the active slip system is determined by minimizing the residual $L_{2}$ norm between the experimental and theoretical components of the in-plane strain tensor [138].

Despite their success, these methods inherently rely on the occurrence of planar and discrete slip and generally assume single slip activity within a given region. As a result, their applicability becomes limited when deformation occurs through diffuse, wavy, or cross-slip, or when multiple slip systems are activated simultaneously. To overcome these constraints, a more generalized, local slip system identification framework termed SSLIP (for slip system-based local identification of plasticity) has recently been proposed [55]. In this method, the experimental displacement gradient tensor,

(14) $$H^{\exp}=\nabla u$$

is directly computed from the measured displacement field obtained by HR-DIC. Assuming small strains and neglecting elastic contributions, the theoretical displacement gradient tensor resulting from slip on all possible systems can be expressed as

(15) $$H^{\mathrm{theor}}=\sum_{\alpha =1}^{N}\gamma^{\alpha }s^{\alpha }⊗n^{\alpha }$$

where $\gamma^{\alpha }$ is the shear amplitude associated with slip system α and N is the total number of slip systems. The identification of the active slip systems and their respective shear contributions is then formulated as a constrained optimization problem, in which the total slip system activity is minimized subject to the condition that the residual difference between the experimental and theoretical displacement gradient tensors remains below a specified threshold $H_{\mathrm{thresh}}$:

(16) $$\underset{\gamma^{\alpha }}{\min}\sum_{\alpha =1}^{N}|\gamma^{\alpha }|\;\mathrm{given}\;||H^{\exp}-H^{\mathrm{theor}}||<H_{\mathrm{thresh}}$$

This optimization framework enables simultaneous consideration of all potential slip systems without assuming planar or single-slip conditions, thereby allowing robust identification even in complex, multi-slip or cross-slip deformation regimes.

**Figure 9.**
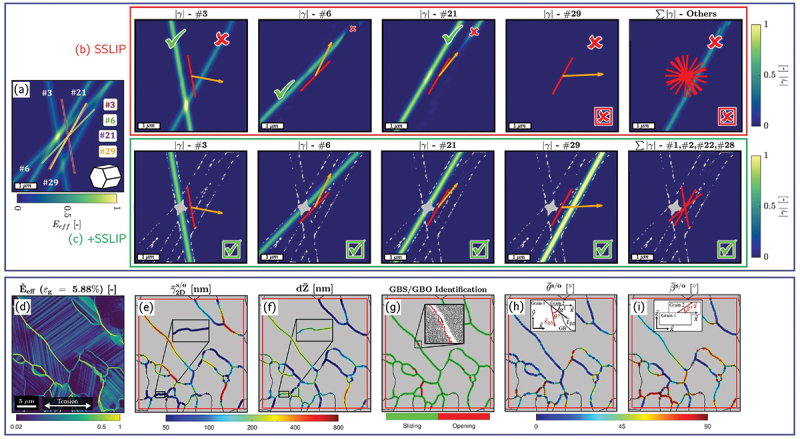
(a) Effective strain field generated from virtual HR-DIC on HCP material and corresponding slip system identified by (b) the SSLIP method and (c) the +SSLIP method. Green checks indicate correctly identified slip systems while red crosses indicate wrong ones (adapted from [176]). (d–i) Grain boundary sliding/opening analysis using the SLIDE method. (d) Effective strain map, (e–f) in-plane and out-of-plane displacement map, (g) grain boundary sliding/opening identification. (h) In-plane angle between the sliding/opening vector and the grain boundary tangent vector. (i) Out-of plane angle between the sliding/opening vector and the tensile direction (adapted from [177]).

The method has been successfully validated on both virtual datasets for HCP materials and experimental strain maps for FCC and body-centered cubic (BCC) alloys, involving up to 48 possible slip systems (Figures 9(a)) [55]. SSLIP was also applied successfully experimentally on HCP materials [103,178]. Furthermore, to enhance computational efficiency and to reduce ambiguity (especially for HCP materials), the procedure was further extended to include a preliminary slip-system filtering step based on Radon transform analysis, which provides an initial estimate of the most likely active slip planes prior to the optimization stage, while also accounting for rotation, and was referred to as +SSLIP [176] (Figure 9(c)). The SSLIP approach was also further enriched to include slip strength anisotropy, hardening evolution laws, and crystallographic reorientation upon straining to improve the slip identification [57].

#### 3.2.2. Grain boundary sliding and opening

Deformation mechanisms at grain boundaries (GBs), including sliding (GBS), opening (GBO), cracking, and migration, are important in materials such as Mg, Zn, ultrafine-grained alloys, and metals deformed at elevated temperature. HR-DIC combined with EBSD provides the spatial resolution needed to examine these processes. One approach involves computing the in-plane (tangential) displacements along the GB, which gives an estimate of the degree of GBS that occurs [100,107,179], which can be extended to 3D by conducting additional optical profilometry [108]. However, reliably separating GB deformation from nearby intragranular slip remains challenging, because the experimental displacement gradients often contain contributions from slip bands, lattice rotations, and noise [177]. To address this, a recent framework termed SLIDE (for sliding identification by local integration of displacements across edges) uses the EBSD-derived GB geometry together with the kinematic definitions of sliding and opening to test whether the measured deformation is compatible with either mechanism [177]. In this approach, theoretical displacement-gradient tensors describing ideal sliding or opening are projected into the DIC plane, and their parameters are optimized to best match the experimental in-plane displacement gradients (Figure 9(d)). Only deformation that satisfies these kinematic constraints is attributed to GBS or GBO, while incompatible components, such as slip-induced shear near the boundary, are rejected. Additionally, the SEM contrast is used to determine opening events. By integrating the identified sliding or opening components along the GB normal direction, the method provides quantitative estimates of in-plane GBS/GBO, and, when available, can be combined with out-of-plane height measurements to reconstruct full 3D GB displacements (Figure 9(f)). This physics-informed approach enables more reliable identification of GB-mediated deformation and offers a path toward studying how sliding and opening interact with intragranular plasticity at the sub-grain scale.

#### 3.2.3. Twinning- and transformation-induced plasticity

In materials with low stacking-fault energy (SFE), such as FCC austenitic steels (*e.g*., 316), several high-entropy alloys, and HCP systems including Mg and Ti alloys, plastic deformation may proceed not only by dislocation slip but also through twinning-induced plasticity (TWIP). Deformation twinning involves the coordinated shear of atoms that produces a mirror-symmetric reorientation of part of the crystal lattice across a specific twinning plane. Unlike slip, which can accumulate gradually, twin formation typically manifests as abrupt strain bursts, pronounced lattice reorientation, and sharp changes in the local mechanical response. Twinning mechanisms play a central role in strain hardening, ductility enhancement, and texture evolution, notably through a dynamic Hall-Petch effect resulting from rapid subdivision of grains.

**Figure 10.**
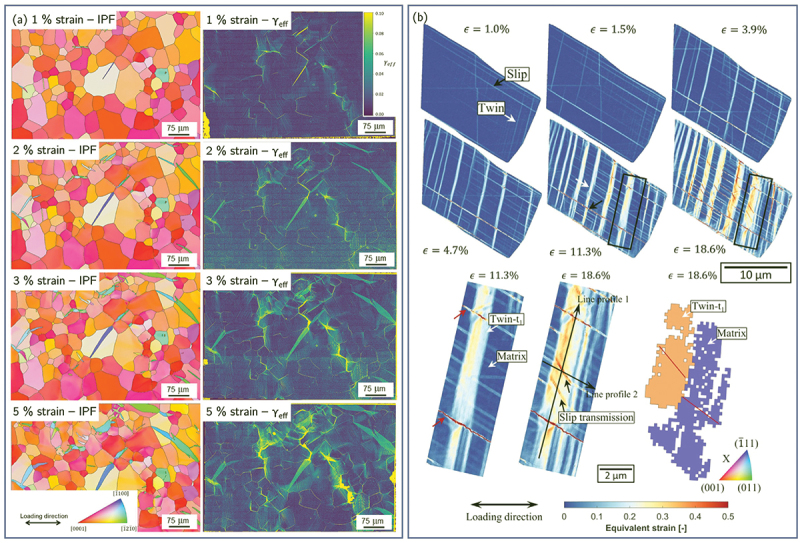
(a) EBSD map and corresponding effective shear strain maps of a pure Mg tensile sample subjected different strain levels revealing the nucleation and propagation of extension twins (adapted from [99], with permission from Elsevier). (b) Equivalent plastic strain maps at different strain levels of a SUS316L grain revealing the sequential occurrence of slip, twin and slip transfer (adapted from [138], with permission from Elsevier).

EBSD has long been the primary tool for studying deformation twinning because the characteristic and sharp 30–90° lattice reorientation provides a clear crystallographic signature [180–182]. However, twin nucleation is strongly influenced by local stress concentrations at grain boundaries, triple junctions, and hard/soft grain interactions, features that EBSD captures only in a static, post-mortem sense. HR-DIC, by contrast, provides access to dynamic twin nucleation, growth, and interaction events, enabling direct visualization of precursor strain localization preceding twin activation [14,59,99,138,183]. HR-DIC studies have shown that twinning often initiates in regions of pronounced deformation incompatibility and is accompanied by highly localized shear bands that interact with grain boundaries and pre-existing slip bands. Recent work in Mg alloys demonstrated that twin variant selection is not predominantly governed by the twinning Schmid factor, but is instead controlled by the compatibility of the twinning shear with neighboring grain boundaries, a factor that cannot be inferred from EBSD alone [99] (Figure 10(a)). Similarly, in FCC stainless steel, the presence of primary slip bands prior to twin nucleation was found to impede subsequent twin propagation, resulting in a heterogeneous strain distribution within the twin. Upon further deformation, slip transfer from the matrix into the twin occurred once the twin band exceeded a critical thickness [138] (Figure 10(b)). Additionally, deformation twinning manifests itself as a characteristic shear strain, which can be identified directly from HR-DIC [184]. The Heaviside-DIC algorithm has also been modified to account for discrete speckle pattern change associated to twin formation by introducing a dead-zone of pixel with a given thickness in the discrete line definition [76].

In steels containing mechanically unstable retained austenite (RA), such as medium-Mn, quenched and partitioned (Q&P), or certain dual-phase microstructures, plastic deformation may also proceed through transformation-induced plasticity (TRIP) such as martensitic transformation, which contributes additional hardening capacity and strongly influences strain partitioning [185]. HR-DIC has emerged as a powerful tool for resolving the nanoscale sequence of slip-assisted transformation events and their interplay with microstructural heterogeneities [96,166]. Recent HR-DIC studies in ultrafine-grained medium-Mn steels show that strain localizes first within austenite grains, where sequential activation of slip and martensitic transformation forms thin high-strain channels that subsequently trigger yielding of the surrounding ferrite and promote Lüders-band propagation [96]. In lath-martensitic Q&P steels, HR-DIC combined with atom probe tomography (APT) reveals that carbon- and Mn-stabilized RA exhibits a strong neighborhood-dependent mechanical response, accommodating high local strains when embedded in tempered martensite, while RA adjacent to fresh martensite or ferrite transforms more readily, thereby modulating strain partitioning and delaying damage initiation [166]. Additionally, HR-DIC has been used to measure the characteristic transformation strain of lath martensite in order to validate new martensite transformation theories [186].

#### 3.2.4. Slip transfer across interfaces

When an incoming dislocation or dislocation array impinges on an interface, several outcomes are possible: it may be blocked, transmitted into the neighboring grain, absorbed and annihilated within the boundary, or absorbed and subsequently re-emitted as a new dislocation on a different slip system [187–189]. Whether slip transfer occurs strongly depends on the nature and morphology of the interface (grain/twin/phase boundary) as well as the geometric alignment between incoming (α) and outgoing (β) slip systems. Slip transfer has major implications for the mechanical performance of polycrystalline metals: dislocation pileup at interfaces can increase local stresses and promote early damage nucleation, whereas efficient transfer reduces local stress concentration. For these reasons, quantitatively assessing slip-boundary interactions remains an active research area aimed at establishing reliable, physics-based criteria for predicting slip transmission.

A wide range of transmission criteria have been proposed and comprehensively reviewed in the literature [187–189]. Many are based on crystallographic alignment metrics that require identifying both the active incoming and potential outgoing slip systems. A commonly used example is the Luster – Morris parameter [190]:

(17) $$m{{\;}^{\prime }}_{\alpha \beta }=|(n^{\alpha }.n^{\beta }).(b^{\alpha }.b^{\beta })|.$$

However, evaluating such criteria reliably is challenging in practice. Conventional slip trace analysis provides information about the active slip plane but not the slip direction, and the common assumption that the slip system with the highest Schmid factor is activated among coplanar systems introduces uncertainty because local stresses are rarely uniform or uniaxial at the grain scale. Consequently, extensive TEM characterization or other high-resolution techniques such as by combining EBSD with electron channeling contrast imaging (ECCI) are often required to confirm the actual slip directions and to provide accurate crystallographic input for slip transfer analyses [191].

**Figure 11.**
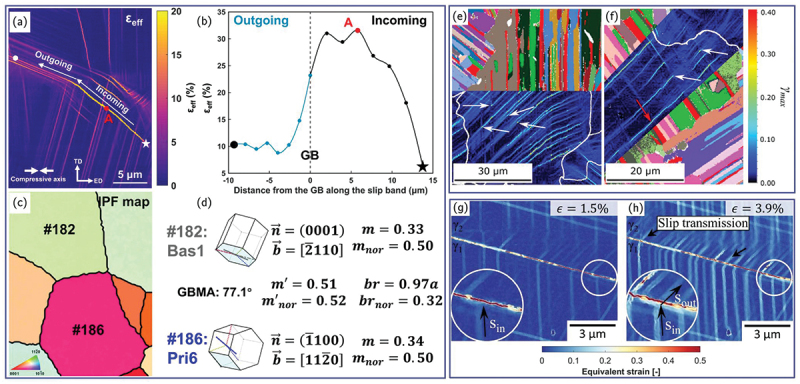
(a–d) Slip transfer across a grain boundary in an extruded Mg-10.6Y alloy. (a) Strain map from HR-DIC, (b) strain profile along the path depicted in (a), (c) corresponding IPF map and (d) calculation of slip transfer parameter (adapted from [58] with permission from Elsevier). (e–f) Slip transfer across lamellae in a γ-TiAl alloy (adapted from [192] with permission from Elsevier). (g–h) Slip transfer across twin boundary in a 316 stainless steel (adapted from [138] with permission from Elsevier).

Instead, HR-DIC combined with EBSD offers a more practical and informative alternative, as it enables not only the identification of active slip systems using the methods described above, but also the direct quantification of the sequence, extent, and effectiveness of slip transfer across individual boundaries (Figure 11). The application of HR-DIC to slip transfer phenomena is still relatively recent [58,96,97,174,192], yet early studies already demonstrate its strong potential to reveal previously unrecognized modes of strain accommodation and intergranular transfer [193]. By capturing the spatio-temporal evolution of slip impingement, blockage, and reactivation across boundaries, HR-DIC provides a powerful new framework for validating and refining slip-transfer criteria in polycrystalline materials.

### 3.3. Data-driven and machine-learning-based feature extraction

HR-DIC has transformed strain-field measurement from a purely experimental tool for locating deformation hotspots into a rich, spatially resolved data modality that captures the physical signatures of microscale deformation mechanisms [194]. By generating large datasets that resolve heterogeneous strain fields at the grain and sub-grain scale, HR-DIC provides an ideal foundation for data-driven and machine-learning (ML) approaches aimed at uncovering relationships between microstructure, local deformation processes, and macroscopic mechanical behavior. Broadly, ML applications for HR-DIC data can be classified into two categories: (1) feature extraction and (2) representation learning and predictive modeling. The present section focuses on the first category while the second category is discussed in Integration with other techniques.

**Figure 12.**
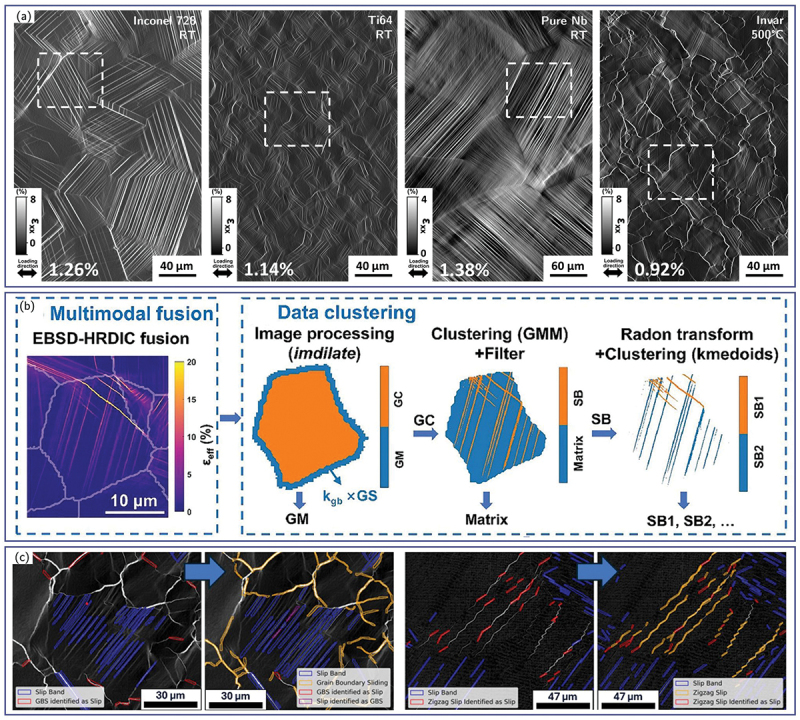
(a) Examples of strain maps along the loading direction for an inconel 718, a Ti-6Al-4 V, a pure niobium, and an Invar specimens deformed to macroscopic plastic strains of 1.26 %, 1.14 %, 1.38 %, and 0.92 %, respectively (adapted from [195] with permission from Elsevier). (b) Data clustering for matrix and slip band based on Gaussian mixture model (adapted from [58] with permission from Elsevier). (c) Detection of deformation events for FCC slip, zigzag slip and GB sliding via computer vision (adapted from [195] with permission from Elsevier).

While previously described physics-based feature-extraction approaches are effective for specific material systems, these approaches often lack robustness when applied across different alloys, deformation modes, or DIC acquisition conditions. As illustrated in Figure 12(a), deformation features can exhibit highly variable morphologies depending on crystal structure, stacking-fault energy, grain size, and loading path, making rule-based segmentation difficult to generalize.

Recent advances in ML-based computer vision, particularly semantic and instance segmentation, have enabled rapid and systematic extraction of deformation events directly from HR-DIC strain maps. An example is shown in Figure 12(b), where ML-driven segmentation combined with EBSD data allows simultaneous identification of microstructural features (*e.g*., GBs) and intragranular deformation events [58]. This integrated approach enables automated quantification of strain partitioning between intergranular and intragranular regions, as well as robust detection of slip bands within individual grains – capabilities that are difficult to achieve using conventional analysis pipelines.

A further example is provided in Fig. 12(c) [195], where a computer-vision model initially trained to detect planar slip bands was progressively extended to identify additional deformation modes, including grain-boundary sliding and wavy or zig-zag slip patterns. This iterative expansion demonstrates the adaptability and transferability of ML-based approaches, highlighting their potential to capture diverse deformation mechanisms with markedly different spatial characteristics using a unified framework.

#### 3.4. Summary

Taken together, the strain localization descriptors discussed above provide complementary, mechanism-specific information rather than a single universal metric. Table 2 synthesizes key strain localization metrics and analysis frameworks used in HR-DIC studies, summarizing their typical domains of applicability, underlying assumptions, and representative references. This overview highlights that metric selection must be guided by both the dominant deformation mechanism and the local microstructural context.

**Table 2.** Summary of key strain localization metrics used in HR-DIC studies, their primary deformation mechanisms, main assumptions or limitations, and representative references.

Metric/approach	Primary deformation mechanism	Main assumptions/limitations	Ref.
$\varepsilon_{\mathrm{eq}}[-]$, $\gamma_{\max}[-]$	Generic strain localization	Scalar descriptors;	[55,97,137–139]
		not mechanism-specific;	
		insensitive to slip direction	
Slip trace analysis	Crystallographic slip	Slip direction not uniquely identified;	[53,58,97,138,139,142,174,175]
		ambiguous for coplanar systems	
Heaviside-DIC	Discrete slip	Requires sharp displacement discontinuities;	[48,51,53,75,140–144]
		limited for diffuse deformation	
RDR	Single/co-planar slip deformation	Assumes planar slip;	[49,52,58,174]
		sensitive to noise and spatial resolution	
SSLIP/+SSLIP	Multi-slip and cross-slip deformation	Small-strain assumption;	[55,57,103,176,178]
		Requires prior slip-plane estimation (+SSLIP)	
SLIDE	Grain boundary sliding and opening	Requires accurate grain-boundary geometry;	[177]
		kinematic assumptions	
Rotation fields, $\omega_{z}$ [∘]	Twin bands and lattice reorientation	Twin formation produces quasi-uniform	[14,137,192]
		rotation bands in DIC	
Slip transfer metrics	Slip transfer across interfaces	Requires accurate multiple slip system identification	[58,96,97,174,192,193]
ML-based feature extraction	Mixed deformation mechanisms	Requires representative training data;	[58,194,195]
		limited physical interpretability	

For slip-dominated deformation, scalar measures such as equivalent plastic strain or maximum shear strain remain effective for identifying localization hotspots, whereas slip-system – resolved approaches derived from HR-DIC (e.g. RDR and SSLIP) enable direct identification of active systems and their relative contributions, including under multi-slip conditions. In twin-dominated materials, localization is more appropriately characterized by abrupt shear offsets, localized strain bursts, and lattice rotation fields associated with discrete twin formation, where HR-DIC provides dynamic information that complements post-mortem EBSD signatures. For grain-boundary-mediated mechanisms such as sliding and opening, conventional strain metrics are often insufficient; instead, kinematically constrained approaches such as SLIDE, which explicitly account for boundary geometry and displacement discontinuities, allow quantitative separation of intergranular deformation from nearby intragranular slip. Finally, analysis of slip transfer across interfaces requires combining slip-system identification with spatially resolved strain and displacement fields to assess both the occurrence and effectiveness of transmission.

Finally, a note on the general limitations of characterizing plasticity using SEM-based measurements. Because HR-DIC fundamentally yields in-plane surface displacement fields (and quantities derived from their spatial gradients), it provides a powerful view of localized plasticity, but remains inherently limited by 3D-to-2D projection. This is particularly relevant for identifying crystallographic slip and grain-boundary sliding/opening (GBS/GBO), where part of the physical displacement and deformation can be oriented out of the specimen plane. In such cases, the in-plane signatures remain highly informative, but may not uniquely capture the full 3D kinematics. This projection aspect helps clarify the scope of different identification strategies: scalar localization measures (e.g. $\varepsilon_{\mathrm{eq}}$ or $\gamma_{\max}$) are highly effective at detecting where deformation concentrates, yet they are intentionally non-specific with respect to the underlying mechanism. Trace-based identification approaches can robustly identify likely slip planes from surface intersections, but care is warranted when distinct slip planes produce similar surface traces (or when multiple systems share comparable trace orientations). Methods that more directly exploit displacement discontinuities, such as Heaviside-DIC or the RDR method, can sharpen slip-system identification when their assumptions hold, but they still operate on the in-plane components and are therefore most discriminating for planar, slip-dominated events with a clear in-plane signature. More general optimization frameworks such as SSLIP/+SSLIP extend identification to diffuse (multi-)slip by fitting the experimentally measured in-plane displacement-gradient tensor, which improves robustness in complex deformation while remaining tied to the 2D information content and associated modeling assumptions (e.g. small-strain kinematics and simplified elastic contributions). In that setting, non-uniqueness can arise because different combinations of slip systems may reproduce very similar in-plane kinematics, and in particular because distinct systems can share the same slip trace and similar projected in-plane slip directions. For interfacial mechanisms, algorithms such as SLIDE similarly leverage the rich in-plane gradients by projecting idealized sliding/opening kinematics into the DIC plane, enabling quantitative in-plane GBS/GBO extraction when the boundary geometry and kinematic assumptions are well satisfied, while full separation of interfacial modes benefits from complementary out-of-plane displacement information. In Laser scanning confocal microscopy (LSCM), we provide strategies to obtain information on out-of-plane deformation.

## 4. Integration with other techniques

### 4.1. In-situ mechanical testing

**Figure 13.**
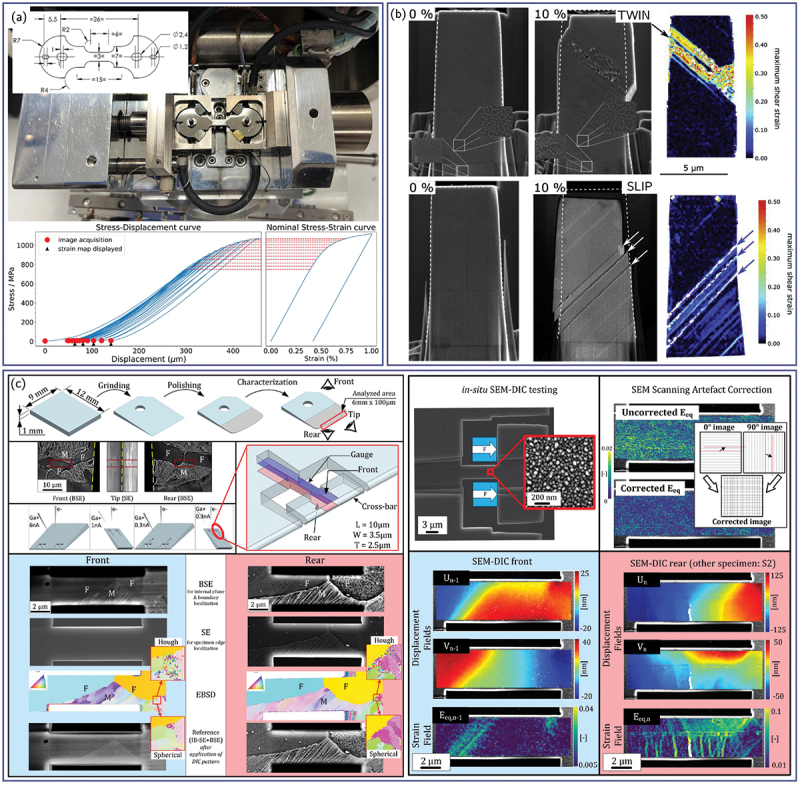
(a) Example of a tensile rig with sample geometry for in-situ tensile testing and corresponding stress-displacement and stress-strain curves (adapted from [196]). (b) Secondary electron images before and after compression of TiAl pillars exhibiting twin and slip, and corresponding maximum shear strain map (adapted from [94]). (c) General overview of sample preparation by FIB, microstructure characterization and front and back sides HR-DIC displacements and strain maps obtained from *in-situ* tensile test (adapted from [69]).

HR-DIC can be implemented either *ex-situ*, by interrupting mechanical tests and (repeatedly) inserting the specimen into an SEM for image acquisition, or (quasi) *in-situ*, by imaging (typically) at intermittent deformation steps while loading of the specimen is paused, using an *in-situ* testing rig inside the microscope. Time-resolved *in-situ* testing is also possible with the condition of a unique region of interest and rather fast image acquisition (few seconds), as demonstrated by the analysis of the slip band propagation during the continuous loading of a Ti-7Al titanium alloy [140]. Further improvements in speed and efficiency may be achieved by multi-beam SEM imaging, capturing up to 91 SEM images simultaneously [194]. *Ex-situ* approaches are experimentally simpler and permit long acquisition times under optimal imaging conditions; however, they inherently suffer from strain relaxation, reverse slip, time-dependent recovery during unloading, and geometrical misalignments, which can hinder (measurement of) the true deformation state. Moreover, small misalignments between scans can introduce phantom rotations which may affect the identification of (lattice) rotations and slip system activities [176]. In-situ mechanical testing inside the SEM overcomes these limitations by enabling strain fields to be measured directly under applied load. Miniaturized tensile, compression, bending, and fatigue rigs integrated into SEM chambers allow controlled mechanical loading over a wide range of forces and temperatures while maintaining high spatial resolution imaging, either by manual acquisition of images at various deformation increments [36,88,102] or by automation [68,100,140,196–200] (Figure 13(a)).

A major experimental challenge for *in-situ* HR-DIC is maintaining image focus and geometric stability during deformation. Changes in specimen height, bending, or rigid-body rotation during loading can induce defocus and apparent displacement artifacts that degrade the signal-to-noise ratio, particularly at high magnification where the depth of field is limited. Moreover, the area of interest typically moves during deformation, requiring manual or automatic repositioning by beam shift or stage movements. These effects are further compounded by the physical size of *in-situ* loading rigs, which typically impose a larger working distance (WD = 10–20 mm) as compared to conventional *ex-situ* SEM imaging (WD = 3–10 mm). An increased working distance typically reduces spatial resolution, electron collection efficiency, beam stability, and depth-of-field control, thereby amplifying image noise and limiting the minimum resolvable subset size for HR-DIC. Mitigation strategies include the use of rigid loading frames, careful specimen alignment, load-hold imaging protocols, and frequent refocusing routines synchronized with image acquisition.

Manual image acquisition, which also requires manual intervention of the aforementioned issues, is time consuming and does not guarantee reproducibility. To achieve statistically representative measurements at high spatial resolution, *in-situ* HR-DIC increasingly relies on automated image acquisition schemes [68,100,140,196–200]. Large regions of interest are commonly captured using tiled image mosaics acquired sequentially at each loading step, followed by image stitching either before or after DIC. Automation of stage motion, imaging parameters, and synchronization with mechanical loading enables reproducible acquisition of hundreds to thousands of high-resolution images during a single experiment [100,196,197,200]. Load – unload – reload protocols can also be employed, particularly for cyclic loading, fatigue, and damage studies [196]. Imaging under full load captures the active deformation state, while intermittent unloading steps provide insight into elastic recovery, residual strain accumulation, and irreversible deformation mechanisms [201].

### 4.2. In-situ micro-mechanical testing

Whilst most applications of HR-DIC have been on macro-scale specimens, a growing number of research groups applies such DIC strain mapping after initial EBSD mapping to micro- and nano-mechanical specimens, in order to augment the opportunities of analysis and interpretation from a single small-scale test: (1) locating and quantifying crystallographic plasticity relative to the microstructure; (2) identifying and quantifying the local occurrence of non-crystallographic plasticity; (3) measuring true strain, and hence stiffness, directly on the specimen to circumvent system compliance determination issues common to small-scale testing; (4) evaluating the coherency of plasticity between multiple faces of oligocrystal specimens. Due to the small specimen sizes, a greater range of materials show plasticity: in addition to just metals and alloys, intermetallics, ceramics, composites of the previous, and even biological materials, have all been the topic of HR-DIC-augmented micromechanical testing.

#### 4.2.1. General considerations

The integration of micro-mechanical testing rigs in SEMs is well established. However, adaptation to HR-DIC requires several considerations: firstly, the geometry should enable unobscured, ideally normal, view of a flat surface of the specimen gage. This is most often achieved by FIB milling of cuboidal gage testpieces either on mechanically or ion polished edges of bulk samples [93,202], or on the end of mechanically or electrochemically-thinned narrow wedges or needles [69]. Such geometries are inherently less mechanically stable than mid-surface locations on the bulk, due to higher lateral compliance, which in the case of edge samples is asymmetric – hence risking outward rotation of a pillar base in compression, or torsional moments along a cantilever aligned with the edge: these can be understood by FEM models [203,204], and partially mitigated, such as by milling free-standing walls at the bulk sample edge [205]. Furthermore, although these geometries lend themselves well to EBSD crystal orientation mapping of the gage, additional FIB polishing at low ion acceleration, *e.g*., at 2 kV, is often required to remove the amorphized surface damage layer resulting from 30 kV milling [39] – equivalently to TEM sample preparation [206]. Secondly, a patterning method (Section 2.1) which can produce a speckle pattern locally, and in a one-step process at room temperature, without liquid contact – to avoid contamination of the small-scale specimens – is required. Additionally, antenna effects of small structures with sharp edges can result in non-uniform thicknesses upon magnetron sputtering [207]. Hence, thus far exploited are: Focused electron beam-induced deposition (FEBID) [39,93,94,192,202,208–213] (Figure 13(b)), direct sputter deposition of InSn [69,103] (Figure 13(c)), and ion milling [214], where the latter is least recommended for small scale testing due to the risk of artificially introducing dislocation sources. Thirdly, the high magnification, single-frame SEM imaging conditions appropriate to micron-scale gage surfaces at the best resolution possible, for lowest-noise DIC, lends itself to the use of a magnetic immersion lens (see Image acquisition and noise sources) and beam monochromator, along with the low working distance necessary for such modes. Whilst this works well for *ex-situ* imaging, *in-situ* nanoindenter geometries can prevent the use of such highest-resolution imaging modes, or an insertable backscatter detector – hence secondary electron imaging at approx. 10 – 15 mm working distance is often used for *in-situ* testing where several images of the surface pattern are acquired throughout loading [192,208]. It can be necessary to adapt the DIC procedure by manually masking to exclude non-gage regions, and also multiple-seeding, depending on the degree of plasticity localization: this is particularly the case for single-crystal and single-lamellar-colony gage sections oriented for single-slip, where the slip displacement along each surface slip line can be many multiples of the subset size [94,209]. Finally, like regular micromechanical testing, such specimen geometries lend themselves well to post-mortem TEM imaging to further interpret details beyond the spatial resolution of HR-DIC [211]; lift-out lamellae may be taken from the near-surface region, or rather from mid-depth, depending on interpretation aims [208,211].

#### 4.2.2. Application examples

HR-DIC analysis of a cuboidal microcompression specimen of Cu oriented for single slip [93] was used as an early example of how crystal lattice rotation can be extracted from HR-DIC datasets as detailed in Computation of strain fields, and this was coherent with EBSD-derived lattice rotation similarly captured in the unloaded state. In a study of a Cr2AlC micropillar [209], also in single slip geometry, the extent of easy basal slip of the MAX phase along each slip line was captured to produce one-to-one correlations of loading curve features and slip events, in order to achieve greater precision in measurement of the resolved shear stress for slip activation by assigning the relevant pillar cross-sections to each loading curve feature – relative scatter was effectively decreased from 52% to just 4%.

Several cases of multiphase alloys have been explored using microcompression; in most cases, the specimen was constituted of a single crystalline parent or matrix phase, to capture plasticity partitioning between the phases: in BCC Fe-Ni2AlTi superalloys [212], a low magnitude of misfit strain was shown to reduce the degree of heterogeneity in plasticity between the phases. Two phase intermetallic γ-TiAl alloys with a lamellar structure were investigated in microcompression (Figure 13(b)) across a range of temperatures up to 700° C [39,94,210,211] and in fatigue up to 10^5^ cycles [192], using *in-situ* SEM micromechanical testing followed by ex situ imaging; in all conditions, the FEBID Pt speckle resisted thermal exposure and repeated imaging well. However, careful tailoring of the low-kV FIB polish was necessary to avoid twin operation tearing any FIB-amorphised surface layer. Such studies revealed extensive operation of the little-reported twinning of the γ phase parallel to the lamellar planes, across all conditions, and early activation of slip activity close and parallel to twin and phase interfaces.

Other mechanical test geometries have received less attention, and only at ambient temperatures, likely due to their greater complexity in specimen preparation and/or alignment for loading. Micro-tensile testing of oligocrystals of dual phase steel [69] (Figure 13(c)) and a Zn alloy [103] with front- and back-side HR-DIC strain mapping has sought to capture the interplay in plasticity across grain and phase boundaries. Strain mapping by both HR-DIC and HR-EBSD (see HR-EBSD mapping) of the near-notch region of a micro-cantilever of tungsten captured toughening slip line emanating from the crack tip, and demonstrated an absence of evidence for such plasticity in the conventional post-mortem TEM imaging of dislocation structures on a near-surface extracted lamella [208].

Two studies, on microtension of a CoCrFeMnNi alloy [214] and micro double shear testing of Si [213], have exploited the good match between SEM view-field for HR-DIC resolution imaging, and the gage dimension for small scale mechanical testing, to perform quasi-continuous imaging during loading, with SEM frame times between 0.5 – 3 s. Data filtering measures were taken to reduce the influence of scan errors from fast imaging on HR-DIC strain results, particularly during initial elastic loading.

Finally, such HR-DIC can also be used to experimentally evaluate otherwise difficult to measure elastic parameters, such as the modulus of pyrolytic graphite (PG) when sandwiched between SiC within a FIBbed micropillar [202]. The effect of a gradient in basal texture (determined by HR-TEM) across the ≈ 1 μm thick PG layer was captured by HR-DIC upon compressing normal to the tri-layer planes. Similarly, HR-DIC has been used on FIBbed micron-scale structures to measure in-plane near-surface residual stress states in coatings, and following surface treatments using the ring milling method [215], among other geometries. Where surface features are insufficient for DIC, a FEBID pattern is usually added, where this must be sufficiently coarse to resist stray FIB-milling. For details, the reader is referred to the extensive NPL Good Practice Guide [216]. In both cases, elastic strain data extraction, and eventually its isolation from plastic strain [202], is the primary focus; therefore, large subset sizes are exploited to reduce strain noise. However, due to the small specimen size (largest dimension < 5 m), high SEM magnifications, commonly 50,000 or higher, the spatial resolution of strain mapping remains below 0.2 m.

### 4.3. Laser scanning confocal microscopy (LSCM)

Although HR-DIC provides quantitative in-plane displacement and strain fields at sub-micron spatial resolution, it offers only indirect access to out-of-plane deformation through the previously described identification methods (Strain localization analysis from HR-DIC results) [51]. Laser scanning confocal microscopy (LSCM) has gained some interest as a way to complement HR-DIC by providing high-resolution topographical maps of the deformed surface [108,136,138,161,217–220]. Unlike atomic force microscopy (AFM), which can resolved nanometer-scales steps but is limited by slow scanning speed, small FOV, and tip-surface interaction artifacts (which could be exacerbated by the speckle pattern), LCSM enables rapid, non-contact imaging over millimeter-size FOV, making it more compatible with HR-DIC mapping.

**Figure 14.**
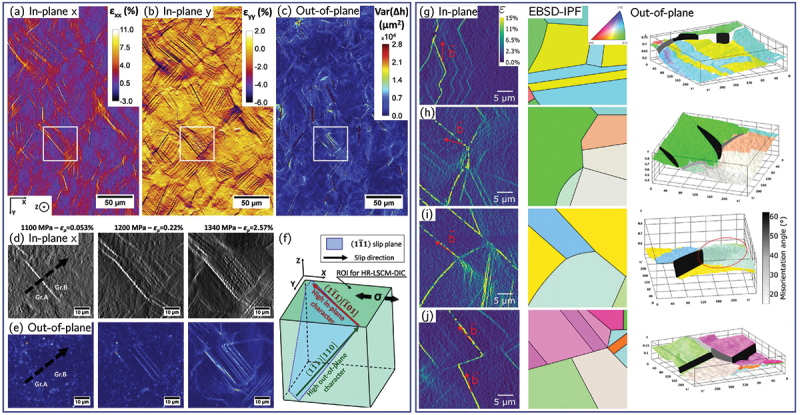
(a–f) Identification of active slip system based on the in-plane and out-of-plane displacements obtained by Heaviside-DIC and LSCM, respectively (adapted from [220], with permission from Elsevier). (g–j) interaction between slip bands and grain/twin boundaries revealed by LSCM-DIC (adapted from [108], with permission from Elsevier).

Early work showed that LSCM can resolve grain-scale and sub-grain-scale roughness arising from orientation-induced incompatibilities [217,218], and subsequent studies demonstrated that combining LSCM height maps with DIC enables simultaneous measurement of in-plane and out-of-plane components of slip-band deformation at the sub-grain scale for the three-dimensional evaluation of the cumulated Burgers vector [220]. LSCM-DIC has proven particularly valuable for identifying active slip systems, because the sign and magnitude of surface steps provide a strong constraint on the Burgers vector projection and thus on the three-dimensional shear direction (Figure 14(a)) [220]. More recently, integrated DIC – LSCM approaches have been used to characterize the three-dimensional configuration of slip and twinning in low-SFE stainless steels and nickel-based superalloys, revealing how nanotwin networks and grain/twin boundaries control the out-of-plane surface relief and generate large local deformation gradients (Figure 14(b)) [108,138]. LSCM-DIC was also used to investigate the out-of-plane character of grain boundary sliding or opening at high temperature [107,108,177]. Contrary to slip localization, grain boundary sliding is not crystallographic but morphological based on the geometry of the grain boundaries and cannot be intuited from in-plane kinematics field. Therefore, topographic measurements are of great importance for the study of grain-boundary events.

### 4.4. HR-EBSD mapping

HR-EBSD enables quantitative measurement of small lattice rotations and elastic strains with a precision several orders of magnitude higher than conventional EBSD. Instead of relying on Hough-based indexing of Kikuchi patterns to determine absolute crystal orientations, HR-EBSD compares diffraction patterns acquired at closely spaced points through cross-correlation similar to DIC. Sub-pixel shifts in Kikuchi band positions encode minute changes in the lattice metric, enabling extraction of the full relative deviatoric elastic strain tensor and lattice rotation tensor with sensitivities on the order of $10^{-4}$ in strain and $10^{-4}$ radians in rotation [66,221–223]. The deviatoric elastic strain tensor provides access to type III stresses, defined as localized intragranular stresses generated by lattice distortions around defects such as dislocations, subgrain structures, or phase boundaries [224]. Meanwhile, the lattice rotation tensor yields a lower-bound estimate of geometrically necessary dislocation (GND) density via Nye’s tensor formalism. When paired with HR-DIC, HR-EBSD becomes a powerful complementary probe: HR-DIC maps total surface deformation and slip localization, while HR-EBSD reveals the underlying elastic strain partitioning, lattice curvature, and dislocation densities that drive heterogeneity in crystalline plasticity.

However, integrating HR-EBSD with HR-DIC poses practical challenges because HR-EBSD requires high-quality, high-contrast Kikuchi patterns, which can be degraded by opaque or thick speckle patterns. Early studies therefore used a sequential workflow in which EBSD was acquired prior to DIC patterning and again post-deformation after speckle removal [225–227]. This limited correlative analysis to a single deformation interruption and prevented simultaneous mapping of the evolving elastic and plastic fields. Recent work has thus focused on developing speckle patterns suitable for HR-DIC while remaining sufficiently electron transparent for HR-EBSD without removal. Several approaches have now demonstrated such dual compatibility, including electron-beam – deposited Pt speckles on W [208], Ag nanoparticle coatings deposited from ethanol suspensions [228,229], and remodeled Ag films formed via NaBr in isopropanol [46]. These advances now enable concurrent HR-DIC/HR-EBSD mapping.

**Figure 15.**
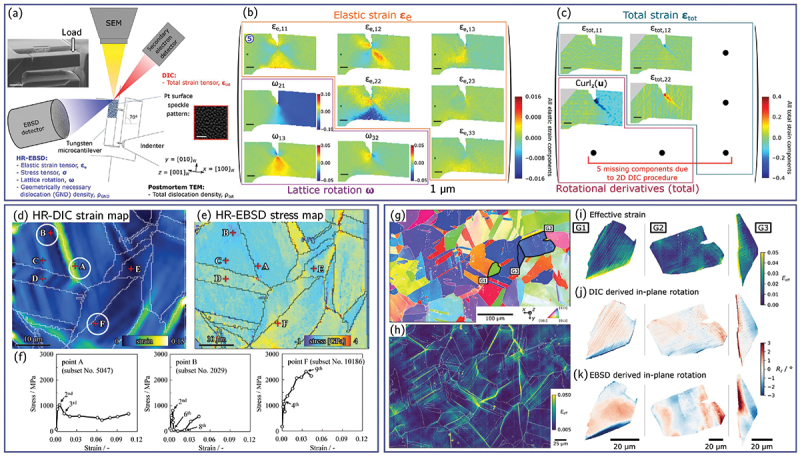
(a–c) Elastic, plastic and total strains estimated around the notch of a W single crystal microcantilever during bending by in situ HR-EBSD and HR-DIC. (a) Schematic of the framework, (b) elastic strain and lattice rotation components map estimated from HR-EBSD and (c) total strain estimated from HR-DIC (adapted from [208]). (d–e) strain and stress map obtained by HR-DIC and HR-EBSD on a deformed nickel-based alloy and (f) examples of extracted microscopic stress-strain curves (adapted from [228], with permission from Elsevier). (g–k) HR-DIC/HR-EBSD study on a CuCrZr alloy revealing connection between strain localization and lattice curvature (adapted from [46], with permission from John Wiley and Sons).

The capability of combined HR-DIC and HR-EBSD is illustrated in Figure 15(a), which shows the deformation of a W microcantilever [208]. HR-DIC captured the nucleation and evolution of discrete slip bands, while HR-EBSD quantified the associated elastic strain and lattice rotation fields. By subtracting the elastic deformation gradient measured via HR-EBSD from the total deformation measured by HR-DIC, the pure plastic deformation gradient can be obtained. This revealed that regions of high elastic stress or high GND density do not necessarily coincide with the areas of largest plastic shear, and that the most active slip band underwent near-complete mechanical annealing. The study demonstrated that dislocation nucleation governs crack-tip plasticity, an insight inaccessible to either technique alone. Another recent example introduces a framework that combines HR-DIC strain mapping with HR-EBSD stress measurement to construct microscopic, spatially resolved stress – strain curves within a polycrystal [228] (Figures 15(d)). By acquiring SEM images and HR-EBSD maps at multiple deformation steps and aligning them through georeferencing, pixel-wise stress – strain responses were extracted, revealing extreme spatial heterogeneity driven by grain orientation, slip activity, and boundary constraints. Lastly, Figure 15(g) shows an application to a deformed CuCrZr alloy [46], where intense HR-DIC slip localization coincides with strong HR-EBSD lattice curvature, directly linking slip band formation to back-stress accumulation and dislocation patterning.

While HR-EBSD is a powerful technique, it should be noted that it yields only relative measures of elastic strain, since a stress-free reference point is required in each grain. This poses no issues for single crystal tests that provide stress-free regions outside the specimen gage [208]. However, polycrystalline specimens should be treated carefully, even when the stress-free patterns are used from the undeformed configuration [228], because pattern center uncertainties may introduce phantom strains [230]. To alleviate these issues, absolute HR-EBSD is under active development in the literature [231–233] and may soon reach the maturity level required for combination with HR-DIC.

### 4.5. Crystal plasticity simulations

Strain mapping obtained from HR-DIC has increasingly been coupled with full-field micromechanical simulations based on CP models. These models typically solve the continuum mechanics boundary value problem using either the finite element method (FEM) or spectral solvers based on the FFT [234–236]. By explicitly representing polycrystalline aggregates incorporating microstructural descriptors such as grain morphology, crystallographic orientation, phase distribution, and grain – grain interactions, full-field CP simulations are capable of computing local intragranular stress and strain fields. This multiscale framework effectively bridges the gap between crystal-level deformation mechanisms and the macroscopic mechanical response of the material, enabling a physics-based interpretation of experimental observations.

Most CP models rely on finite-strain kinematics, in which the total deformation gradient F is multiplicatively decomposed into an elastic part $F_{e}$ and a plastic part $F_{p}$ [237]:

(18) $$F=F_{e}F_{p}.$$

Assuming plastic deformation occurs solely by crystallographic slip, the plastic velocity gradient is

(19) $$L_{p}={\dot{F}}_{p}F_{p}-1=\sum_{\alpha =1}^{N}{\dot{\gamma }}^{\alpha }s^{\alpha }⊗n^{\alpha },$$

where ${\dot{\gamma }}^{\alpha }$ is the shear rate on slip system α, expressed as a function of the resolved shear stress $\tau^{\alpha }=S:(s^{\alpha }⊗n^{\alpha })$ and a set of internal state variables (ISVs):

(20) $${\dot{\gamma }}^{\alpha }=f(\tau^{\alpha },\mathrm{ISVs}).$$

Different CP formulations vary in their choice of ISVs (*e.g*., CRSS, dislocation densities) and the constitutive evolution laws that govern them, leading to models that range from highly phenomenological to strongly physics-based [234,238].

A major challenge lies in the parameter calibration and validation of CP models. The large number of constitutive parameters, associated with the various deformation mechanisms, hardening laws, and grain-boundary interactions, introduces a high degree of non-uniqueness in the model response. Calibration solely against macroscopic stress – strain curves can therefore be misleading, as different parameter sets may reproduce the global mechanical behavior equally well without correctly capturing the underlying micro-mechanical processes and deformation heterogeneity at the microstructure scale. In this regard, full-field experimental strain maps obtained from HR-DIC provide a powerful validation pathway, offering spatially resolved data against which local predictions from CP models can be rigorously tested. Conversely, CP simulations can serve as a ‘virtual laboratory/digital twin’ offering mechanistic insights that complement HR-DIC measurements and extend their interpretive power. Global DIC is for instance a slip based-regularization method aiming at identifying CP parameters from surface in-plane kinematics fields in the case diffuse plastic fields [50]. Conventional CPFEM calculations provide diffuse plastic fields that do not correspond to the discrete and heterogeneous slip localization observed using HR-DIC. Therefore, FFT-based strain gradient plasticity 3D polycrystalline simulations are better suited to depict slip localization on 3D aggregates [239,240].

#### 4.5.1. General framework

**Figure 16.**
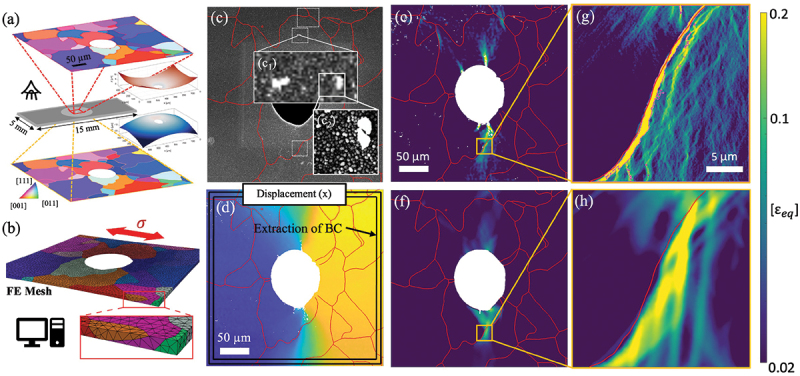
General overview of a quasi-2D integrated DIC-CPFE study. (a) EBSD characterization and thickness measurement through optical profilometry on both sides of the specimen. (b) 3D microstructure generation and conformal meshing. (c) Speckle pattern at various scales. (d) Extraction of boundary conditions from displacement fields. Comparison of mesoscale equivalent plastic strain fields obtained by (e) DIC and (f) CPFE simulations. Insets of a small area, comparing (f) HR-DIC strain fields and (g) CPFE simulations with discrete slip bands (adapted from [241]).

Figure 16 summarizes an integrated experimental – numerical workflow for combining HR-DIC measurements with CP simulations. It is important to emphasize that the framework described here represents a recent and still emerging approach, rather than a routine methodology. In many published studies, only subsets of these steps are implemented. The workflow presented below should therefore be regarded as an idealized reference framework, illustrating best practices and highlighting recent methodological advances that aim to maximize the fidelity of experiment – simulation coupling.

The polycrystalline geometry used as input for CP simulations is typically reconstructed from 2D EBSD maps from surface characterization. Because EBSD provides only surface information, the subsurface morphology is unknown. The common assumption that the microstructure remains constant through thickness, producing columnar extrusions of surface grains, can introduce significant error, particularly when the true 3D grain geometry influences surface stress and strain fields [83,141,242,243]. Several advances have been proposed to overcome this limitation. Quasi-2D approaches involving fabrication of micrometer-thin foils and EBSD mapping on both sides [241,244] provide near-surface volumetric constraints (Figure 16(a)). More sophisticated serial-sectioning techniques, such as TriBeam tomography [245–247], allow full 3D reconstruction, enabling direct use of experimentally determined microstructures in CPFE simulations [141,248]. Although experimentally demanding, these methods represent promising avenues toward reducing geometrical uncertainty in experiment-numerical comparisons. Such 3D characterization offer high spatial resolution and angular resolution, making possible grain reorientation study in the volume [141]. Knowing the 3D morphology and orientation of grains also aims to better understand and correlate surface slip intensity to microstructural features in the volume, such as triple junction [141]. Non-destructive techniques such as diffraction contrast tomography (DCT) are capable to reconstruct 3D aggregates both in morphology and grain orientation for further HR-DIC or CPFEM calculations [161,249,250].

Following EBSD acquisition, the dataset undergoes cleaning, distortion correction, and grain identification before mesh generation. Two meshing strategies are common. Structured meshing on a regular grid is straightforward but produces staircase grain boundaries and uniform element density. Unstructured conformal meshing, on the other hand, follows the true grain boundary morphology and allows adaptive refinement, improving fidelity and computational efficiency [241,251–254] (Figure 16(b)).

Boundary conditions (BCs) critically influence the agreement between simulation and experiment. BCs can be implemented as prescribed displacements or applied tractions [40,60,90,254–256]. Alternatively, submodeling approaches interpolate BCs from a larger-scale model to a detailed microstructural zone [226]. More recently, HR-DIC has enabled direct extraction of BCs from measured displacement fields, which can be interpolated or extrapolated onto the simulation boundaries [183,219,241,248,257,258] (Figure 16(d)). This methodology provides a realistic coupling between experiment and simulation, as it captures the actual deformation gradients imposed on the observed region, including any heterogeneities arising from neighboring microstructural features outside the FOV. However, such fully prescribed BCs can also be too strict, enforcing noise and systematic errors onto the simulations [259], which can be alleviated by imposing ‘relaxed’ BCs [244].

#### 4.5.2. Application examples

**Figure 17.**
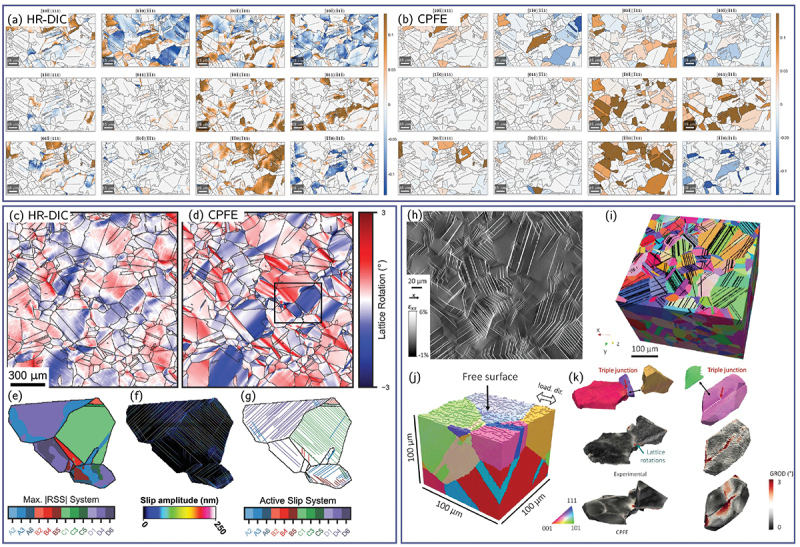
(a–b) Comparison of slip activity obtained from (a) HR-DIC measurements and (b) CP simulations in a strained high-purity copper sample (adapted from [57]). (c–g) Correlative analysis of deformation in an In718 alloy: (c) lattice rotation measured by HR-DIC and (d) lattice rotation predicted by CP simulations; (e) resolved shear stress (RSS) fields from CP simulations; (f) slip amplitudes extracted by Heaviside-DIC; and (g) the corresponding identified active slip systems (adapted from [254], with permission from Elsevier). (h–k) Integrated HR-DIC/CPFE study on deformed In718: (h) surface strain field measured by HR-DIC; (i) reconstructed 3D microstructure from TriBeam serial sectioning and EBSD with overlaid slip traces; (j) finite element mesh generated directly from the reconstructed microstructure; and (k) comparison of lattice rotation fields measured by EBSD and predicted by CP simulations (adapted from [141], with permission from Elsevier).

Conventional calibration methods that rely solely on fitting macroscopic stress – strain curves often lead to significant uncertainty, as multiple sets of CRSS values can reproduce the same global mechanical response. Calibration of CP parameters based on HR-DIC strain fields can resolve ambiguities inherent to macroscopic fitting. Spatially resolved comparisons between measured and simulated strain localization, slip activity, or lattice rotation allow discrimination between competing CRSS ratios and deformation mechanisms [50,57,59,159,175,183,256,258,260] (Figures 17(a)).

Beyond calibration, HR-DIC observations have directly motivated refinement of CP models. In lath martensitic steels, early activation of habit-plane slip or interfacial sliding has been incorporated either by modifying CRSS values [60,90] or by introducing constitutive mixtures of martensite and retained austenite with restricted active slip systems [102,241]. These enriched models substantially improve agreement with experimental strain maps (Figures 16(e)).

Validated CP simulations can then be used predictively to analyze strain localization, damage initiation, and crack formation [40,67,141,170,171,226,254,261–263]. Comparison of predicted lattice rotation and resolved shear stresses with HR-DIC and HR-EBSD measurements (Figures 17(c)) demonstrates that CPFE accurately captures lattice curvature while revealing intragranular stress heterogeneities that drive variant selection and multisystem slip. Finally, combined HR-DIC and 3D EBSD studies have shown that integrated simulation – experiment frameworks can statistically relate slip localization to grain-boundary character and triple junction geometry, with CPFE explaining the underlying stress fields that govern planar slip vs. curved multislip behavior (Figures 17(h)) [141].

#### 4.5.3. Limitations and challenges

Beyond the aforementioned limitations associated with the use of two-dimensional microstructures in most correlated HR-DIC/CP studies, several fundamental challenges remain. A major limitation arises from the inherently different nature of the experimental and numerical representations of deformation. One-to-one comparisons between HR-DIC strain maps and CP simulation results generally yield good qualitative and quantitative agreement when relatively large subset sizes and spacings are employed in the DIC analysis, producing smooth and continuous strain fields [50,59,67,170,171,183,260]. Consequently, the discrete and highly localized features observed experimentally, such as individual slip lines, micro-bands, or twin lamellae, typically cannot be directly reproduced by conventional continuum-based CP formulations.

To overcome this mismatch, several studies have proposed methods to mimic strain localization phenomena within CP simulations. These approaches typically involve the manual or algorithmic introduction of discrete slip or twin bands, in which localized softening is imposed to simulate intense shear deformation. The inclusion of such bands can produce strain patterns that visually resemble the experimentally observed slip bands [60,90,239–241,244,264–266]. However, these methods introduce additional modeling complexity and are often accompanied by numerical challenges. In particular, the strong mesh-size dependence of the localized deformation and the emergence of numerical instabilities can compromise the robustness and physical interpretability of the results. Non-local or gradient-enhanced formulations, which introduce intrinsic length scales into the constitutive framework may regularize strain localization and reduce mesh sensitivity [239,240,267].

### 4.6. Data-driven and machine-learning approaches

#### 4.6.1. Representation learning

**Figure 18.**
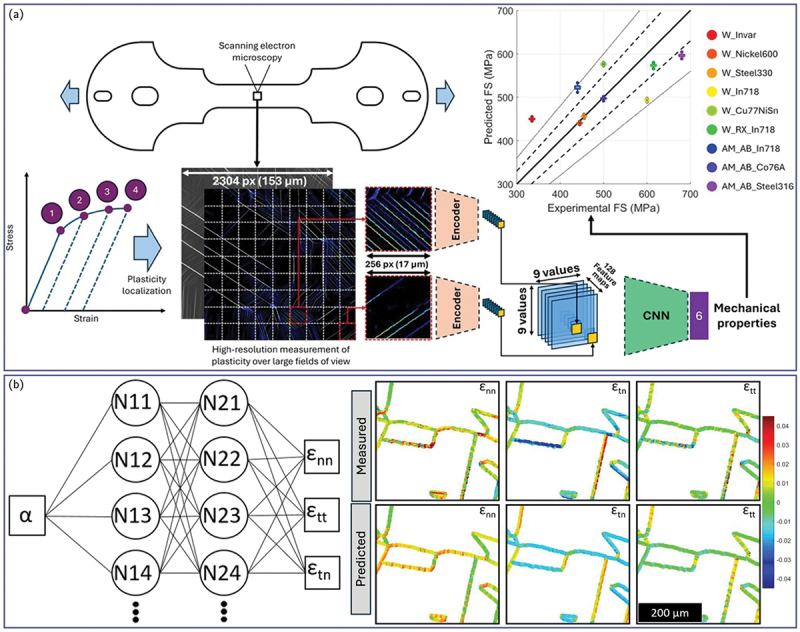
(a) Mechanical property prediction via plasticity encoding and mapping using variational autoencoder (adapted from [269] with permission from Elsevier). (b) Measured and predicted grain boundary strains by HR-DIC and combined neural network (adapted from [270] with permission from Springer Nature).

Because HR-DIC data contains rich information on the evolution of plasticity during loading, they can be used to inform mechanical properties through conventional materials-science approaches, either by fundamentally investigating the links between microstructure, plastic deformation, and macroscopic mechanical behavior, or by calibrating and validating numerical models that relate local strain and stress fields to mechanical properties. Significant work in the literature has explored these classical approaches. For example, relationships between the characteristics of slip events and the fatigue ratio (fatigue strength divided by yield strength) have been established [143] and subsequently leveraged to rapidly predict fatigue properties directly from HR-DIC data [268], a task that is typically time-consuming using conventional fatigue testing (Figure 18(a)). In parallel, the combination of CP simulations calibrated or validated using HR-DIC has yielded significant insights into the role of microstructure in governing deformation and has been used to inform mechanical properties [52,85,137,170,183,236,263,271].

#### 4.6.2. Predictive modeling

Beyond traditional microstructural descriptors, strain fields provide a powerful complementary modality for predicting mechanical properties. Because these full-field measurements encode how a material actually distributes and localizes deformation under load, they capture microstructure – mechanics interactions that are often missing from purely structural or chemical descriptors. Incorporating strain data, as latent representations learned through ML models, augments conventional material descriptors and enables more accurate prediction of mechanical macroscopic properties such fatigue resistance [272], or fracture behavior. This fusion of microstructural information with experimentally measured deformation fields creates a richer, mechanistically informed feature space that substantially improves predictive performance. While conventional DIC (optical DIC) provides limited information on discrete deformation mechanisms, HR-DIC offers complementary insights by directly capturing discrete deformation events such as slip, grain boundary sliding or opening or deformation twinning. As a result, HR-DIC is theoretically much better suited to enrich and complete conventional material descriptors. However, only limited work currently exists in this area, though rapid progress in HR-DIC automation and advances in deep-learning methodologies will likely accelerate developments.

Representative examples already illustrate this potential. For instance, neural-network models trained directly on HR-DIC strain fields aligned with EBSD data have been used to predict where plastic strain accumulates near grain boundaries in polycrystalline metals using only microstructural descriptors, most notably the local grain-boundary orientation relative to the loading axis [270] (Figure 18(b)). By learning correlations between HR-DIC – measured strain fields and EBSD-derived geometry, the model successfully reproduced spatial trends and local extrema of grain-boundary strain, highlighting the potential of strain-informed ML to identify microstructural drivers of damage-prone regions. Although limited to a reduced set of input features, this work illustrates how HR-DIC can supply physically meaningful training data for predictive models targeting fatigue- and creep-relevant strain localization phenomena.

The relatively recent and slow development of ML approaches applied to HR-DIC data primarily stems from the intrinsic complexity of these datasets, which are both spatial, in the form of high-resolution strain maps, and temporal, as sequences of maps acquired at successive macroscopic deformation levels. Encoding such rich spatiotemporal information requires sophisticated ML architectures and, consequently, large and representative training datasets. While the generation of such datasets is increasingly feasible through high-throughput and automated HR-DIC methodologies, the establishment of sufficiently large legacy datasets remains time-consuming, a challenge that is well recognized by the community. In addition, the effectiveness and generality of ML architectures applied to HR-DIC data, particularly when combined with complementary modalities such as EBSD, remain only partially explored. Variational autoencoders have demonstrated promising capabilities for encoding HR-DIC strain fields and extracting meaningful latent representations [269], yet the robustness of these latent spaces with respect to training set size, material class, and loading path is still insufficiently understood. In particular, questions remain regarding the transferability of learned representations across different microstructures, materials, and deformation conditions, as well as the influence of the multiplicity of strain fields that can be obtained from a single microstructure. Despite these current limitations, significant opportunities exist. Ongoing developments in ML toward spatial intelligence and world-model architectures are particularly well suited to capturing the structured, multiscale, and multimodal nature of HR-DIC data and its combination with microstructural characterization techniques such as EBSD. When coupled with the continued expansion of large, high-quality datasets enabled by automation and high-throughput experimentation, these approaches are expected not only to improve direct property inference from HR-DIC data, but more importantly to enable a transition from empirical correlations toward ML-guided identification of governing deformation mechanisms. At present, physically interpretable ML frameworks applied to HR-DIC remain in their infancy, with only limited examples leveraging attention-based or explainable architectures to identify controlling plasticity features directly from strain-field data. However, the integration of such interpretability-driven approaches with high-fidelity, large-field-of-view HR-DIC and EBSD measurements holds strong potential to establish a new paradigm for alloy design guided by ML spatial intelligence.

## 5. Summary and perspectives

HR-DIC extends conventional DIC into the sub-micron regime by exploiting SEM imaging and appropriately scaled speckle patterns, enabling direct visualization of intragranular strain localization in bulk metallic materials. When combined with optimized imaging conditions and careful noise control, HR-DIC provides quantitative displacement and strain fields that resolve individual deformation mechanisms, thereby bridging the gap between TEM-scale mechanism identification and macroscopic mechanical response.

The accuracy and interpretive power of HR-DIC critically depend on speckle pattern design and image acquisition strategies. Patterning methods such as thin-film remodeling, particle deposition, and etching-based approaches now routinely provide nanometric, high-contrast speckles suitable for HR-DIC at sub-grain scales. However, the smallest subset sizes reported for HR-DIC rarely reache 100 nm or lower [14,94,96,102]. With modern SEMs capable of stable imaging at spatial resolutions of 1 nm and below, it should be feasible to further reduce the speckle sizes to 5 nm and lower, to enable HR-DIC with subset sizes below 50 nm. The challenge is to create a pattern that is small enough, while still yielding sufficient contrast for SEM imaging. Recent application to in-TEM nanomechanical testing [64] to study the grain boundary sliding of olivine along an amorphous layer arising from stress-induced amorphisation, increased the resolution of DIC to a subset size of just 6 nm. Additionally, while routinely performed under controlled environmental conditions (*e.g*., ambient temperature and low humidity), HR-DIC studies performed under extreme environments remain limited. In particular, experiments at elevated temperature or in reactive atmospheres pose major challenges, as surface oxidation or corrosion progressively alters the speckle morphology required for reliable image correlation. To date, high-temperature DIC experiments conducted in air have been restricted either to short exposure times [115,273] or to temperatures sufficiently low to limit oxide growth [106,192,211]. At higher temperatures, even oxide layers thinner than about 200 nm can severely degrade HR-DIC performance. The resulting changes in surface topography and electron opacity make reliable correlation impossible beyond approximately 650° C in air [106,192,211]. In contrast, *in-situ* and *ex-situ* HR-DIC under SEM has been successfully demonstrated at temperatures up to about 750 $\;{\;}^{\circ }$ C in inert or secondary-vacuum environments, where oxidation is suppressed [107–109,113,135,274]. Nevertheless, these conditions remain far from representative of many service environments relevant to high-temperature alloys. Developing speckle patterns that retain contrast, adhesion, and morphological stability over extended durations under oxidizing or corrosive conditions therefore remains a major open challenge. Addressing this limitation will be essential for extending HR-DIC toward realistic thermo-mechanical testing and for enabling quantitative, time-resolved studies of creep, oxidation-assisted damage, and environmentally driven strain localization such as in irradiated environment [77,116,117,275].

HR-DIC calculations based on conventional subset-based formulations, while well optimized, remain computationally demanding, even for FOV on the order of a few hundred micrometers. Automated image acquisition and analysis over specimen-scale regions rapidly generate massive datasets, for which DIC processing, even when accelerated using GPUs, can require substantial computational time and resources [194]. In addition, the post-processing and interpretation of local deformation mechanisms using the methodologies described in Strain localization analysis from HR-DIC results are often slow, susceptible to user-dependent bias, and may fail to extract all relevant features from complex strain fields. Together, these limitations have motivated an emerging paradigm in DIC based on hybrid, data-driven learning frameworks. In such approaches, classical DIC principles, such as correlation metrics, subset matching, and physical constraints, are embedded within deep neural network architectures. This integration significantly improves robustness, reduces training-data requirements, and enables accurate displacement and strain estimation even under challenging speckle or noise conditions. Similarly, the automated features extraction and reduction in dimensionality of the data by ML, may open the door to accelerated material design and properties predictions [269,276].

Beyond localized deformation, an emerging paradigm in metallic materials design focuses on strain delocalization as a route to overcome the strength – ductility trade-off. Recent studies emphasize that mechanical performance is governed not only by the initiation of localized strain, but by how effectively strain can be redistributed across multiple length scales through heterogeneous microstructures, including gradients, lamellae, nanotwins, and hierarchical architectures. Advanced *in-situ* strain characterization techniques, such as HR-DIC, are uniquely positioned to quantify this delocalization by tracking the spatio-temporal evolution of strain from elastic lattice distortion to plastic flow and damage accumulation [277]. Coupling HR-DIC with (HR)-EBSD, and micromechanical simulations therefore provides a powerful experimental foundation for identifying microstructural design principles that promote stable plastic flow, enhanced work hardening, and delayed failure. Looking forward, integrating HR-DIC with data-driven analysis and multiscale modeling will be essential for translating the concept of strain delocalization into predictive design strategies for next-generation high-performance metallic materials [278].

## References

[1] Mughrabi H. Dislocation wall and cell structures and long-range internal stresses in deformed metal crystals. Acta Metallurgica. 1983;31(9):1367–43. doi: 10.1016/0001-6160(83)90007-X

[2] Fressengeas C, Molinari A. Instability and localization of plastic flow in shear at high strain rates. J The Mech Phys Solids. 1987;35(2):185–211. doi: 10.1016/0022-5096(87)90035-4

[3] Kubin L, Estrin Y. Evolution of dislocation densities and the critical conditions for the Portevin-Le Châtelier effect. Acta Metallurgica et Materialia. 1990;38(5):697–708. doi: 10.1016/0956-7151(90)90021-8

[4] Lubliner J. Plasticity theory. Dover Publications; 2008. Available from: https://books.google.fr/books?id=MkK-BLbHtcAC

[5] François D, Pineau A, Zaoui A. Mechanical behaviour of materials: volume 1: micro- and macroscopic constitutive behaviour. Springer Netherlands; 2012. Available from 10.1007/978-94-007-2546-1

[6] Pineau A, Benzerga A, Pardoen T. Failure of metals i: brittle and ductile fracture. Acta Materialia. 2016;107:424–483. doi: 10.1016/j.actamat.2015.12.034

[7] Pineau A, McDowell DL, Busso EP, et al. Failure of metals II: fatigue. Acta Materialia. 2016;107:484–507. doi: 10.1016/j.actamat.2015.05.050

[8] Peters WH, Ranson WF. Digital imaging techniques in experimental stress analysis. Optical Eng. 1982;21(3). doi: 10.1117/12.7972925

[9] Sutton M, Wolters W, Peters W, et al. Determination of displacements using an improved digital correlation method. Image Vision Comput. 1983;1(3):133–139. doi: 10.1016/0262-8856(83)90064-1

[10] Chu TC, Ranson WF, Sutton MA. Applications of digital-image-correlation techniques to experimental mechanics. Exp Mech. 1985;25(3):232–244. doi: 10.1007/BF02325092

[11] Liu M, Wang J, Yao D, et al. Applications and challenges of digital image correlation in the microscopic environment. Meas Sci Technol. 2025;36(10):102002. doi: 10.1088/1361-6501/adf986

[12] Wang L, Zhang Y, Zeng Z, et al. Tracking the sliding of grain boundaries at the atomic scale. Science. 2022;375(6586):1261–1265. doi: 10.1126/science.abm2612 35298254

[13] Robinson A, Homer ER, Thompson GB. Application of digital image correlation for in-situ deformation studies using transmission electron microscopy. Scr Materialia. 2024;252:116253. doi: 10.1016/j.scriptamat.2024.116253

[14] Orozco-Caballero A, Lunt D, Robson JD, et al. How magnesium accommodates local deformation incompatibility: a high-resolution digital image correlation study. Acta Materialia. 2017;133:367–379. doi: 10.1016/j.actamat.2017.05.040

[15] Kang J, Oh HS, Wei S, et al. An in situ study of microstructural strain localization and damage evolution in an (alpha+beta) Ti-Al-V-Fe-Si-O alloy. Acta Materialia. 2023;242:118424. doi: 10.1016/j.actamat.2022.118424

[16] Briffod F, Shiraiwa T, Enoki M. Micromechanical investigation of the effect of the crystal orientation on the local deformation path and ductile void nucleation in dual-phase steels. Mater Sci Eng: A. 2021;826:141933. doi: 10.1016/j.msea.2021.141933

[17] Hwang S, Kato H, Okada K, et al. Exploring unusual Lüders deformation in ultrafine-grained high-Mn austenitic steel. Mater Res Lett. 2024;12(8):571–579. doi: 10.1080/21663831.2024.2359611

[18] Sutton M, Zhao W, McNeill S, et al. Local crack closure measurements: development of a measurement system using computer vision and a far-field microscope. ASTM International 100 Barr Harbor Drive; 1999. p. 145–156. doi: 10.1520/STP15755S, PO Box C700, West Conshohocken (PA) 19428–2959.

[19] Quinta da Fonseca J, Mummery PM, Withers PJ. Full-field strain mapping by optical correlation of micrographs acquired during deformation. J Microsc. 2005;218(1):9–21. doi: 10.1111/j.1365-2818.2005.01461.x 15817059

[20] Zhao Z, Ramesh M, Raabe D, et al. Investigation of three-dimensional aspects of grain-scale plastic surface deformation of an aluminum oligocrystal. Int J Plasticity. 2008;24(12):2278–2297. doi: 10.1016/j.ijplas.2008.01.002

[21] Roux S, Réthoré J, Hild F. Digital image correlation and fracture: an advanced technique for estimating stress intensity factors of 2D and 3D cracks. J Phys D Appl Phys. 2009;42(21):214004. doi: 10.1088/0022-3727/42/21/214004

[22] Carroll J, Abuzaid W, Lambros J, et al. An experimental methodology to relate local strain to microstructural texture. Rev Sci Instruments. 2010;81(8). doi: 10.1063/1.3474902 20815609

[23] Abuzaid WZ, Sangid MD, Carroll JD, et al. Slip transfer and plastic strain accumulation across grain boundaries in Hastelloy X. J The Mech Phys Solids. 2012;60(6):1201–1220. doi: 10.1016/j.jmps.2012.02.001

[24] Texier D, Palchoudhary A, Genée J, et al. Effect of oxygen dissolution on the mechanical behavior of thin Ti-6Al-4V specimens oxidized at high temperature: experimental and modeling approach. Corros Sci. 2024;235:112177. doi: 10.1016/j.corsci.2024.112177

[25] El Bartali A, Aubin V, Degallaix S. Fatigue damage analysis in a duplex stainless steel by digital image correlation technique. Fatigue Fract Eng Mater Struct. 2008;31(2):137–151. doi: 10.1111/j.1460-2695.2007.01207.x

[26] Efstathiou C, Sehitoglu H, Lambros J. Multiscale strain measurements of plastically deforming polycrystalline titanium: role of deformation heterogeneities. Int J Plasticity. 2010;26(1):93–106. doi: 10.1016/j.ijplas.2009.04.006

[27] Quanjin M, Rejab M, Halim Q, et al. Experimental investigation of the tensile test using digital image correlation (DIC) method. In: Materials Today: Proceedings; Hyderabad, India; 2020. Vol. 27. p. 757–763. doi: 10.1016/j.matpr.2019.12.072

[28] Mao W, Gao S, Gong W, et al. Quantitatively evaluating the huge lüders band deformation in an ultrafine grain stainless steel by combining in situ neutron diffraction and digital image correlation analysis. Scr Materialia. 2023;235:115642. doi: 10.1016/j.scriptamat.2023.115642

[29] Pan B, Qian K, Xie H, et al. Two-dimensional digital image correlation for in-plane displacement and strain measurement: a review. Meas Sci Technol. 2009;20(6):062001. doi: 10.1088/0957-0233/20/6/062001

[30] Hild F, Roux S. Digital image correlation: from displacement measurement to identification of elastic properties – a review. Strain. 2006;42(2):69–80. doi: 10.1111/j.1475-1305.2006.00258.x

[31] Doumalin P, Bornert M. Micromechanical applications of digital image correlation techniques. 2000. doi: 10.1007/978-3-319-51439-0_34 Springer Berlin Heidelberg.

[32] Lagattu F, Bridier F, Villechaise P, et al. In-plane strain measurements on a microscopic scale by coupling digital image correlation and an in situ sem technique. Mater Charact. 2006;56(1):10–18. doi: 10.1016/j.matchar.2005.08.004

[33] Sutton MA, Li N, Joy DC, et al. Scanning electron microscopy for quantitative small and large deformation measurements part i: sem imaging at magnifications from 200 to 10, 000. Exp Mech. 2007;47(6):775–787. doi: 10.1007/s11340-007-9042-z

[34] Sutton MA, Li N, Garcia D, et al. Scanning electron microscopy for quantitative small and large deformation measurements part ii: experimental validation for magnifications from 200 to 10, 000. Exp Mech. 2007;47(6):789–804. doi: 10.1007/s11340-007-9041-0

[35] Di Gioacchino F, Quinta da Fonseca J. Plastic strain mapping with sub-micron resolution using digital image correlation. Exp Mech. 2012;53(5):743–754. doi: 10.1007/s11340-012-9685-2

[36] Kammers AD, Daly S. Digital image correlation under scanning electron microscopy: methodology and validation. Exp Mech. 2013;53(9):1743–1761. doi: 10.1007/s11340-013-9782-x

[37] Vanderesse N, Lagacé M, Bridier F, et al. An open source software for the measurement of deformation fields by means of digital image correlation. Microsc Microanal. 2013;19(S2):820–821. doi: 10.1017/S1431927613006090

[38] Stinville J, Echlin M, Texier D, et al. Sub-grain scale digital image correlation by electron microscopy for polycrystalline materials during elastic and plastic deformation. Exp Mech. 2015;56(2):197–216. doi: 10.1007/s11340-015-0083-4

[39] Edwards TEJ, Di Gioacchino F, Muñoz-Moreno R, et al. Deformation of lamellar TiAl alloys by longitudinal twinning. Scr Materialia. 2016;118:46–50. doi: 10.1016/j.scriptamat.2016.03.004

[40] Guan Y, Chen B, Zou J, et al. Crystal plasticity modelling and hr-dic measurement of slip activation and strain localization in single and oligo-crystal ni alloys under fatigue. Int J Plasticity. 2017;88:70–88. doi: 10.1016/j.ijplas.2016.10.001

[41] Kammers AD, Daly S. Small-scale patterning methods for digital image correlation under scanning electron microscopy. Meas Sci Technol. 2011;22(12):125501. doi: 10.1088/0957-0233/22/12/125501

[42] Hoefnagels J, van Maris M, Vermeij T. One-step deposition of nano-to-micron-scalable, high-quality digital image correlation patterns for high-strain in-situ multi-microscopy testing. Strain. 2019;55(6). doi: 10.1111/str.12330

[43] Klavzer N, Gayot SF, Coulombier M, et al. Nanoscale digital image correlation at elementary fibre/matrix level in polymer–based composites. Composites Part A: Applied Science and Manufacturing. 2023;168:107455. doi: 10.1016/j.compositesa.2023.107455

[44] Dimov N, Weisz-Patrault D, Tanguy A, et al. Strain and damage analysis using high resolution digital image correlation in the stir zone of an AA6061-AA7075 dissimilar friction stir weld. Mater Today Commun. 2023;34:105359. doi: 10.1016/j.mtcomm.2023.105359

[45] Montgomery C, Koohbor B, Sottos N. A robust patterning technique for electron microscopy-based digital image correlation at sub-micron resolutions. Exp Mech. 2019;59(7):1063–1073. doi: 10.1007/s11340-019-00487-2

[46] Poole B, Marsh A, Lunt D, et al. Nanoscale speckle patterning for combined high-resolution strain and orientation mapping of environmentally sensitive materials. Strain. 2024;60(6). doi: 10.1111/str.12477

[47] Edwards T, Di Gioacchino F, Springbett H, et al. Stable speckle patterns for nano-scale strain mapping up to 700°C. Exp Mech. 2017;57(9):1469–1482. doi: 10.1007/s11340-017-0317-8 30930472 PMC6407731

[48] Nie Z, Wang F, Li J, et al. Strain localization induced by closely spaced lamellae structure in a Mg alloy containing long period stacking ordered structure. Int J Plasticity. 2025;193:104423. doi: 10.1016/j.ijplas.2025.104423

[49] Chen Z, Daly SH. Active slip system identification in polycrystalline metals by digital image correlation (DIC). Exp Mech. 2016;57(1):115–127. doi: 10.1007/s11340-016-0217-3

[50] Guery A, Hild F, Latourte F, et al. Slip activities in polycrystals determined by coupling dic measurements with crystal plasticity calculations. Int J Plasticity. 2016;81:249–266. doi: 10.1016/j.ijplas.2016.01.008

[51] Bourdin F, Stinville J, Echlin M, et al. Measurements of plastic localization by heaviside-digital image correlation. Acta Materialia. 2018;157:307–325. doi: 10.1016/j.actamat.2018.07.013

[52] Xu X, Lunt D, Thomas R, et al. Identification of active slip mode in a hexagonal material by correlative scanning electron microscopy. Acta Materialia. 2019;175:376–393. doi: 10.1016/j.actamat.2019.06.024

[53] Charpagne M, Stinville J, Callahan P, et al. Automated and quantitative analysis of plastic strain localization via multi-modal data recombination. Mater Charact. 2020;163:110245. doi: 10.1016/j.matchar.2020.110245

[54] Sperry R, Han S, Chen Z, et al. Comparison of EBSD, DIC, AFM, and ECCI for active slip system identification in deformed Ti-7Al. Mater Charact. 2021;173:110941. doi: 10.1016/j.matchar.2021.110941

[55] Vermeij T, Peerlings R, Geers M, et al. Automated identification of slip system activity fields from digital image correlation data. Acta Materialia. 2023;243:118502. doi: 10.1016/j.actamat.2022.118502

[56] León-Cázares FD, Rowlands B, Galindo-Nava EI. Quantification of slip band distribution in polycrystals: an automated fast Fourier transform decomposition approach. Microscopy Microanal. 2023;29(2):580–595. doi: 10.1093/micmic/ozad002 37749723

[57] Depriester D, Jp G, Barrallier L. Slip identification from HR-DIC/EBSD: incorporating crystal plasticity constitutive laws. Int J Solids Struct. 2024;305:113077. doi: 10.1016/j.ijsolstr.2024.113077

[58] Ni R, Boehlert CJ, Zeng Y, et al. Automated analysis framework of strain partitioning and deformation mechanisms via multimodal fusion and computer vision. Int J Plasticity. 2024;182:104119. doi: 10.1016/j.ijplas.2024.104119

[59] Ganesan S, Yaghoobi M, Githens A, et al. The effects of heat treatment on the response of WE43 Mg alloy: crystal plasticity finite element simulation and SEM-DIC experiment. Int J Plasticity. 2021;137:102917. doi: 10.1016/j.ijplas.2020.102917

[60] Briffod F, Shiraiwa T, Yamazaki K, et al. Integrated experimental–numerical investigation of strain partitioning and damage initiation in a low-carbon lath martensitic steel. Mater Sci Eng: A. 2023;876:145148. doi: 10.1016/j.msea.2023.145148

[61] Zhang Y, Dillon S, Lambros J. Creep characterization of amorphous SiO2 in the transmission electron microscope using digital image correlation and finite element analysis. Exp Mech. 2023;63(4):621–636. doi: 10.1007/s11340-022-00937-4

[62] Baral P, Kashiwar A, Coulombier M, et al. Grain boundary-mediated plasticity in aluminum films unraveled by a statistical approach combining nano-DIC and ACOM-TEM. Acta Materialia. 2024;276:120081. doi: 10.1016/j.actamat.2024.120081

[63] Gautier R, Mompiou F, Renk O, et al. Quantifying grain boundary deformation mechanisms in small-grained metals. Nature. 2025;648(8093):327–332. doi: 10.1038/s41586-025-09800-7 41372639

[64] Haq IU, Cordier P, Furstoss J, et al. Stress-induced amorphization promotes grain boundary sliding in olivine. Acta Materialia. 2026;303:121697. doi: 10.1016/j.actamat.2025.121697

[65] Sun Y, Liu J, Blom D, et al. Atomic-scale imaging correlation on the deformation and sensing mechanisms of SnO2 nanowires. Appl Phys Lett. 2014;105(24). doi: 10.1063/1.4904912

[66] Wilkinson AJ, Meaden G, Dingley DJ. High-resolution elastic strain measurement from electron backscatter diffraction patterns: new levels of sensitivity. Ultramicroscopy. 2006;106(4–5):307–313. doi: 10.1016/j.ultramic.2005.10.001 16324788

[67] Fujita N, Ishikawa N, Roters F, et al. Experimental–numerical study on strain and stress partitioning in bainitic steels with martensite–austenite constituents. Int J Plasticity. 2018;104:39–53. doi: 10.1016/j.ijplas.2018.01.012

[68] Chen Z, Lenthe W, Stinville JC, et al. High-resolution deformation mapping across large fields of view using scanning electron microscopy and digital image correlation. Exp Mech. 2018;58(9):1407–1421. doi: 10.1007/s11340-018-0419-y

[69] Vermeij T, Verstijnen J, Ramirez y Cantador T, et al. A nanomechanical testing framework yielding front&rear-sided, high-resolution, microstructure-correlated SEM-DIC strain fields. Exp Mech. 2022;62(9):1625–1646. doi: 10.1007/s11340-022-00884-0

[70] Kang J, Cem Tasan C. Beyond the dynamic Hall-Petch effect: mechanical twinning for microscopic strain delocalization. Scr Materialia. 2025;255:116346. doi: 10.1016/j.scriptamat.2024.116346

[71] Jin H, Bruck H. Pointwise digital image correlation using genetic algorithms. Exp Techniques. 2005;29(1):36–39. doi: 10.1111/j.1747-1567.2005.tb00202.x

[72] Poissant J, Barthelat F. A novel “subset splitting” procedure for digital image correlation on discontinuous displacement fields. Exp Mech. 2009;50(3):353–364. doi: 10.1007/s11340-009-9220-2

[73] Moës N, Belytschko T. Extended finite element method for cohesive crack growth. Eng Fract Mech. 2002;69(7):813–833. doi: 10.1016/S0013-7944(01)00128-X

[74] Réthoré J, Hild F, Roux S. Shear-band capturing using a multiscale extended digital image correlation technique. Comput Methods Appl Mech Eng. 2007;196(49–52):5016–5030. doi: 10.1016/j.cma.2007.06.019

[75] Valle V, Hedan S, Cosenza P, et al. Digital image correlation development for the study of materials including multiple crossing cracks. Exp Mech. 2014;55(2):379–391. doi: 10.1007/s11340-014-9948-1

[76] Anjaria D, Heczko M, Black RL, et al. Plastic deformation delocalization at cryogenic temperatures in a nickel-based superalloy. Acta Materialia. 2024;276:120106. doi: 10.1016/j.actamat.2024.120106

[77] Dong Y, Pan B. A review of speckle pattern fabrication and assessment for digital image correlation. Exp Mech. 2017;57(8):1161–1181. doi: 10.1007/s11340-017-0283-1

[78] Tong W. Detection of plastic deformation patterns in a binary aluminum alloy. Exp Mech. 1997;37(4):452–459. doi: 10.1007/BF02317313

[79] Zhang N, Tong W. An experimental study on grain deformation and interactions in an Al-0.5 mar. Int J Plasticity. 2004;20(3):523–542. doi: 10.1016/S0749-6419(03)00100-1

[80] Jin H, Lu WY, Haldar S, et al. Microscale characterization of granular deformation near a crack tip. J Mater Sci. 2011;46(20):6596–6602. doi: 10.1007/s10853-011-5608-3

[81] Jin H, Lu WY, Korellis J. Micro-scale deformation measurement using the digital image correlation technique and scanning electron microscope imaging. The J Strain Anal Eng Des. 2008;43(8):719–728. doi: 10.1243/03093247JSA412

[82] Hoc T, Rey C. Effect of the free surface on strain localization in mild steel. Scr Materialia. 2000;42(11):1053–1058. doi: 10.1016/S1359-6462(00)00337-7

[83] Héripré E, Dexet M, Crépin J, et al. Coupling between experimental measurements and polycrystal finite element calculations for micromechanical study of metallic materials. Int J Plasticity. 2007;23(9):1512–1539. doi: 10.1016/j.ijplas.2007.01.009

[84] Dimanov A, El Sabbagh A, Raphanel J, et al. Deformation of aluminum investigated by digital image correlation: evidence of simultaneous crystal slip and grain boundary sliding. Metall Mater Trans A. 2024;55(6):1814–1835. doi: 10.1007/s11661-024-07349-0

[85] Tatschl A, Kolednik O. On the experimental characterization of crystal plasticity in polycrystals. Mater Sci Eng: A. 2003;356(1–2):447–463. doi: 10.1016/S0921-5093(03)00095-9

[86] Tschopp M, Bartha B, Porter W, et al. Microstructure-dependent local strain behavior in polycrystals through in-situ scanning electron microscope tensile experiments. Metall Mater Trans A. 2009;40(10):2363–2368. doi: 10.1007/s11661-009-9938-6

[87] Kammers AD, Daly S. Self-assembled nanoparticle surface patterning for improved digital image correlation in a scanning electron microscope. Exp Mech. 2013;53(8):1333–1341. doi: 10.1007/s11340-013-9734-5

[88] Yan D, Tasan CC, Raabe D. High resolution in situ mapping of microstrain and microstructure evolution reveals damage resistance criteria in dual phase steels. Acta Materialia. 2015;96:399–409. doi: 10.1016/j.actamat.2015.05.038

[89] Shafqat S, Hoefnagels JPM. Cool, dry, nano-scale dic patterning of delicate, heterogeneous, non-planar specimens by micro-mist nebulization. Exp Mech. 2021;61(6):917–937. doi: 10.1007/s11340-020-00686-2

[90] Briffod F, Hu H, Shiraiwa T, et al. Effect of in-lath slip strength on the strain partitioning in a dual-phase steel investigated by high-resolution digital image correlation and crystal plasticity simulations. Mater Sci Eng: A. 2023;862:144413. doi: 10.1016/j.msea.2022.144413

[91] Kursun E, Supreeti S, Janssens K, et al. High optical contrast nanoimprinted speckle patterns for digital image correlation analysis. Micro Nano Eng. 2022;17:100164. doi: 10.1016/j.mne.2022.100164

[92] Cannon AH, Hochhalter JD, Bomarito GF, et al. Micro speckle stamping: high contrast, no basecoat, repeatable, well-adhered. River Publishers; 2025. doi: 10.1007/978-3-319-51439-0_34

[93] Di Gioacchino F, Clegg WJ. Mapping deformation in small-scale testing. Acta Materialia. 2014;78:103–113. doi: 10.1016/j.actamat.2014.06.033

[94] Edwards TEJ, Di Gioacchino F, Mohanty G, et al. Longitudinal twinning in a TIAL alloy at high temperature by in situ microcompression. Acta Materialia. 2018;148:202–215. doi: 10.1016/j.actamat.2018.01.007

[95] Bourcier M, Bornert M, Dimanov A, et al. Multiscale experimental investigation of crystal plasticity and grain boundary sliding in synthetic halite using digital image correlation. JGR Solid Earth. 2013;118(2):511–526. doi: 10.1002/jgrb.50065

[96] Yin W, Briffod F, Hu H, et al. Quantitative investigation of strain partitioning and failure mechanism in ultrafine grained medium Mn steel through high resolution digital image correlation. Scr Materialia. 2023;229:115386. doi: 10.1016/j.scriptamat.2023.115386

[97] Hu H, Briffod F, Shiraiwa T, et al. Automated slip system identification and strain analysis framework using high-resolution digital image correlation data: application to a bimodal Ti-6Al-4V alloy. Int J Plasticity. 2023;166:103618. doi: 10.1016/j.ijplas.2023.103618

[98] Luo Y, Ruff J, Ray R, et al. Vapor-assisted remodeling of thin gold films. Chem Mater. 2005;17(20):5014–5023. doi: 10.1021/cm051127w

[99] Sarebanzadeh M, Orozco-Caballero A, Nieto-Valeiras E, et al. Twin nucleation at grain boundaries in Mg analyzed through in situ electron backscatter diffraction and high-resolution digital image correlation. Acta Materialia. 2025;285:120678. doi: 10.1016/j.actamat.2024.120678

[100] Yavuzyegit B, Avcu E, Smith AD, et al. Mapping plastic deformation mechanisms in AZ31 magnesium alloy at the nanoscale. Acta Materialia. 2023;250:118876. doi: 10.1016/j.actamat.2023.118876

[101] Shah K, Sockalingam S, O’Brien H, et al. Sub-microscale speckle pattern creation on single carbon fibers for scanning electron microscope-digital image correlation (SEM-DIC) experiments. Composites Part A. Appl Sci Manuf. 2023;165:107331. doi: 10.1016/j.compositesa.2022.107331

[102] Vermeij T, Mornout C, Rezazadeh V, et al. Martensite plasticity and damage competition in dual-phase steel: a micromechanical experimental–numerical study. Acta Materialia. 2023;254:119020. doi: 10.1016/j.actamat.2023.119020

[103] König D, Vermeij T, Maresca F, et al. Direct comparison of nanoscale plasticity in single and bi-crystal tensile tests extracted from a zinc coating. Mater Sci Eng: A. 2025;932:148128. doi: 10.1016/j.msea.2025.148128

[104] Glushko O, Pippan R, Şopu D, et al. How to catch a shear band and explain plasticity of metallic glasses with continuum mechanics. Nat Commun. 2024;15(1). doi: 10.1038/s41467-024-49829-2 PMC11222483 38961099

[105] Carter JL, Uchic MD, Mills MJ. Impact of speckle pattern parameters on DIC strain resolution calculated from in-situ SEM experiments. River Publishers; 2015. doi: 10.1007/978-3-319-06977-7_16

[106] Stinville JC, Echlin MP, Callahan PG, et al. Measurement of strain localization resulting from monotonic and cyclic loading at 650° C in nickel base superalloys. Exp Mech. 2017;57(8):1289–1309. doi: 10.1007/s11340-017-0286-y

[107] Texier D, Milanese J, Jullien M, et al. Strain localization in the alloy 718 Ni-based superalloy: from room temperature to 650° C. Acta Materialia. 2024;268:119759. doi: 10.1016/j.actamat.2024.119759

[108] Jullien M, Black R, Stinville J, et al. Grain size effect on strain localization, slip-grain boundary interaction and damage in the alloy 718 Ni-based superalloy at 650°C. Mater Sci Eng: A. 2024;912:146927. doi: 10.1016/j.msea.2024.146927

[109] Jullien M, Black RL, Stinville JC, et al. Strain rate effect on strain localization in alloy 718 Ni-based superalloy at intermediate temperature. Springer Nat Switz. 2024:278–286. doi: 10.1007/978-3-031-63937-1_26

[110] Soula A, Renollet Y, Boivin D, et al. Analysis of high-temperature creep deformation in a polycrystalline nickel-base superalloy. Mater Sci Eng: A. 2009;510–511:301–306. doi: 10.1016/j.msea.2008.04.122

[111] Soula A, Locq D, Boivin D, et al. Quantitative evaluation of high temperature deformation mechanisms: a specific microgrid extensometry technique coupled with EBSD analysis. J Mater Sci. 2010;45(20):5649–5659. doi: 10.1007/s10853-010-4630-1

[112] Walley JL, Wheeler R, Uchic MD, et al. In-situ mechanical testing for characterizing strain localization during deformation at elevated temperatures. Exp Mech. 2011;52(4):405–416. doi: 10.1007/s11340-011-9499-7

[113] Carter J, Zhou N, Sosa J, et al. Characterization of strain accumulation at grain boundaries of nickel-based superalloys. Superalloys. 2012. doi: 10.1002/9781118516430.ch5

[114] Carter JL, Kuper MW, Uchic MD, et al. Characterization of localized deformation near grain boundaries of superalloy René-104 at elevated temperature. Mater Sci Eng: A. 2014;605:127–136. doi: 10.1016/j.msea.2014.03.048

[115] Martin G, Caldemaison D, Bornert M, et al. Characterization of the high temperature strain partitioning in duplex steels. Exp Mech. 2012;53(2):205–215. doi: 10.1007/s11340-012-9628-y

[116] Lunt D, Thomas R, Bowden D, et al. Detecting irradiation-induced strain localisation on the microstructural level by means of high-resolution digital image correlation. J Nucl Mater. 2023;580:154410. doi: 10.1016/j.jnucmat.2023.154410

[117] Thomas R, Lunt D, Atkinson M, et al. The role of hydrides and precipitates on the strain localisation behaviour in a zirconium alloy. Acta Materialia. 2023;261:119327. doi: 10.1016/j.actamat.2023.119327

[118] Pan B, Xie H, Wang Z, et al. Study on subset size selection in digital image correlation for speckle patterns. Opt Express. 2008;16(10):7037. doi: 10.1364/OE.16.007037 18545407

[119] Pan B, Lu Z, Xie H. Mean intensity gradient: an effective global parameter for quality assessment of the speckle patterns used in digital image correlation. Optics Lasers Eng. 2010;48(4):469–477. doi: 10.1016/j.optlaseng.2009.08.010

[120] Yu H, Guo R, Xia H, et al. Application of the mean intensity of the second derivative in evaluating the speckle patterns in digital image correlation. Optics Lasers Eng. 2014;60:32–37. doi: 10.1016/j.optlaseng.2014.03.015

[121] Hua T, Xie H, Wang S, et al. Evaluation of the quality of a speckle pattern in the digital image correlation method by mean subset fluctuation. Optics Laser Technol. 2011;43(1):9–13. doi: 10.1016/j.optlastec.2010.04.010

[122] Yaofeng S, Pang JH. Study of optimal subset size in digital image correlation of speckle pattern images. Optics Lasers Eng. 2007;45(9):967–974. doi: 10.1016/j.optlaseng.2007.01.012

[123] Timischl F, Date M, Nemoto S. A statistical model of signal–noise in scanning electron microscopy. Scanning. 2011;34(3):137–144. doi: 10.1002/sca.20282 21898458

[124] Jung KO, Kim SJ, Kim DH. An approach to reducing the distortion caused by vibration in scanning electron microscope images. Nuclear instruments and methods in physics research section A: accelerators, spectrometers. Detectors Associated Equip. 2012;676:5–17. doi: 10.1016/j.nima.2012.01.061

[125] Postek MT, Vladár AE. Does your SEM really tell the truth?—How would you know? Part 1. Scanning. 2013;35(6):355–361. doi: 10.1002/sca.21075 23427011

[126] Postek MT, Vladár AE, Purushotham KP. Does your SEM really tell the truth? How would you know? Part 2. Scanning. 2013;36(3):347–355. doi: 10.1002/sca.21124 24166540

[127] Postek MT, Vladár AE, Cizmar P. Nanomanufacturing concerns about measurements made in the sem part iii: vibration and drift. In: Postek MT, editor. Instrumentation, metrology, and standards for nanomanufacturing, optics, and semiconductors viii. Vol. 9173. SPIE; 2014. p. 917306. doi: 10.1117/12.2062032

[128] Postek MT, Vladár AE. Nanomanufacturing concerns about measurements made in the sem part iv: charging and its mitigation. In: Campo EM, Dobisz EA Eldada LA, editors. Nanoengineering: fabrication, properties, optics, and devices xii. Vol. 9556. SPIE; 2015. p. 95560Q. doi: 10.1117/12.2186997 PMC5486226 28663664

[129] Postek MT, Vladár AE. Nanomanufacturing concerns about measurements made in the sem part v: dealing with noise. In: Campo EM, Dobisz EA Eldada LA, editors. Nanoengineering: Fabrication, properties, optics, and devices XIII. Vol. 9927. SPIE; 2016. p. 99270G. doi: 10.1117/12.2236478

[130] Maraghechi S, Hoefnagels JP, Peerlings RH, et al. Correction of scan line shift artifacts in scanning electron microscopy: an extended digital image correlation framework. Ultramicroscopy. 2018;187:144–163. doi: 10.1016/j.ultramic.2018.01.002 29499524

[131] Mello AW, Book TA, Nicolas A, et al. Distortion correction protocol for digital image correlation after scanning electron microscopy: emphasis on long duration and ex-situ experiments. Exp Mech. 2017;57(9):1395–1409. doi: 10.1007/s11340-017-0303-1

[132] Maraghechi S, Hoefnagels JPM, Peerlings RHJ, et al. Correction of scanning electron microscope imaging artifacts in a novel digital image correlation framework. Exp Mech. 2019;59(4):489–516. doi: 10.1007/s11340-018-00469-w 31205321 PMC6541586

[133] Lenthe WC, Stinville JC, Echlin MP, et al. Advanced detector signal acquisition and electron beam scanning for high resolution SEM imaging. Ultramicroscopy. 2018;195:93–100. doi: 10.1016/j.ultramic.2018.08.025 30216796

[134] Lunt D, Thomas R, Roy M, et al. Comparison of sub-grain scale digital image correlation calculated using commercial and open-source software packages. Mater Charact. 2020;163:110271. doi: 10.1016/j.matchar.2020.110271

[135] Mello AW, Nicolas A, Sangid MD. Fatigue strain mapping via digital image correlation for Ni-based superalloys: the role of thermal activation on cube slip. Mater Sci Eng: A. 2017;695:332–341. doi: 10.1016/j.msea.2017.04.002

[136] Rouwane A, Texier D, Périé JN, et al. High resolution and large field of view imaging using a stitching procedure coupled with distortion corrections. Optics Amp Laser Technol. 2024;177:111165. doi: 10.1016/j.optlastec.2024.111165

[137] Di Gioacchino F, Quinta da Fonseca J. An experimental study of the polycrystalline plasticity of austenitic stainless steel. Int J Plasticity. 2015;74:92–109. doi: 10.1016/j.ijplas.2015.05.012

[138] Yin W, Briffod F, Hu H, et al. Three-dimensional configuration of crystal plasticity in stainless steel assessed by high resolution digital image correlation and confocal microscopy. Int J Plasticity. 2023;170:103762. doi: 10.1016/j.ijplas.2023.103762

[139] Hu H, Briffod F, Yin W, et al. Quantitative investigation of slip band activities in a bimodal titanium alloy under pure fatigue and dwell-fatigue loadings. Int J Fatigue. 2024;182:108203. doi: 10.1016/j.ijfatigue.2024.108203

[140] Stinville J, Francis T, Polonsky A, et al. Time-resolved digital image correlation in the scanning electron microscope for analysis of time-dependent mechanisms. Exp Mech. 2020;61(2):331–348. doi: 10.1007/s11340-020-00632-2

[141] Charpagne M, Hestroffer J, Polonsky A, et al. Slip localization in Inconel 718: a three-dimensional and statistical perspective. Acta Materialia. 2021;215:117037. doi: 10.1016/j.actamat.2021.117037

[142] Charpagne M, Stinville J, Wang F, et al. Orientation dependent plastic localization in the refractory high entropy alloy hfnbtatizr at room temperature. Mater Sci Eng: A. 2022;848:143291. doi: 10.1016/j.msea.2022.143291

[143] Stinville JC, Charpagne MA, Cervellon A, et al. On the origins of fatigue strength in crystalline metallic materials. Science. 2022;377(6610):1065–1071. doi: 10.1126/science.abn0392 36048948

[144] Ahmadikia B, Shadle D, Stinville JC, et al. Role of microstructure on the development of local orientation gradients in polycrystals. J Mater Res Technol. 2024;33:168–179. doi: 10.1016/j.jmrt.2024.08.195

[145] Liberzon A, Käufer T, Bauer A, et al. Openpiv/openpiv-python: openpiv-python v0.23.4; 2021. doi: 10.5281/zenodo.4409178

[146] Stinville JC, Lenthe WC, Echlin MP, et al. Microstructural statistics for fatigue crack initiation in polycrystalline nickel-base superalloys. Int J Fract. 2017;208(1–2):221–240. doi: 10.1007/s10704-017-0241-z

[147] Das YB, Forsey AN, Simm TH, et al. In situ observation of strain and phase transformation in plastically deformed 301 austenitic stainless steel. Mater amp Des. 2016;112:107–116. doi: 10.1016/j.matdes.2016.09.057

[148] Blaber J, Adair B, Antoniou A. Ncorr: open-source 2D digital image correlation MATLAB software. Exp Mech. 2015;55(6):1105–1122. doi: 10.1007/s11340-015-0009-1

[149] Belloni V, Ravanelli R, Nascetti A, et al. Py2dic: a new free and open source software for displacement and strain measurements in the field of experimental mechanics. Sensors. 2019;19(18):3832. doi: 10.3390/s19183832 31491860 PMC6766978

[150] Boukhtache S, Abdelouahab K, Bahou A, et al. A lightweight convolutional neural network as an alternative to dic to measure in-plane displacement fields. Optics Lasers Eng. 2023;161:107367. doi: 10.1016/j.optlaseng.2022.107367

[151] Wang Y, Zhao J. Dic-net: upgrade the performance of traditional DIC with Hermite dataset and convolution neural network. Optics Lasers Eng. 2023;160:107278. doi: 10.1016/j.optlaseng.2022.107278

[152] Dosovitskiy A, Fischer P, Ilg E, et al. Flownet: learning optical flow with convolutional networks. In: 2015 IEEE International Conference on Computer Vision (ICCV); Santiago, Chile. IEEE; 2015; p. 2758–2766. doi: 10.1109/ICCV.2015.316

[153] Duan X, Xu H, Dong R, et al. Digital image correlation based on convolutional neural networks. Optics Lasers Eng. 2023;160:107234. doi: 10.1016/j.optlaseng.2022.107234

[154] Yang R, Li Y, Zeng D, et al. Deep dic: deep learning-based digital image correlation for end-to-end displacement and strain measurement. J Mater Process Technol. 2022;302:117474. doi: 10.1016/j.jmatprotec.2021.117474

[155] Yang J, Qian K, Wang L. R3-dicnet: an end-to-end recursive residual refinement dic network for larger deformation measurement. Opt Express. 2023;32(1):907. doi: 10.1364/OE.505655 38175112

[156] Cheng X, Zhou S, Xing T, et al. Solving digital image correlation with neural networks constrained by strain-displacement relations. Opt Express. 2023;31(3):3865. doi: 10.1364/OE.475232 36785369

[157] Gao J, Wang G, Wang P, et al. Improving deep learning-based digital image correlation with zero mean normalized cross-correlation. Exp Mech. 2025;66(2):237–252. doi: 10.1007/s11340-025-01231-9

[158] Stinville J, Charpagne M, Bourdin F, et al. Measurement of elastic and rotation fields during irreversible deformation using heaviside-digital image correlation. Mater Charact. 2020;169:110600. doi: 10.1016/j.matchar.2020.110600

[159] Goulmy J, Depriester D, Guittonneau F, et al. Mechanical behavior of polycrystals: coupled in situ DIC-EBSD analysis of pure copper under tensile test. Mater Charact. 2022;194:112322. doi: 10.1016/j.matchar.2022.112322

[160] Vermeij T, Hoefnagels J. Plasticity, localization, and damage in ferritic-pearlitic steel studied by nanoscale digital image correlation. Scr Materialia. 2022;208:114327. doi: 10.1016/j.scriptamat.2021.114327

[161] Damas Resende P, Texier D, Genée J, et al. Slip localization and grain boundary sliding analysis at sub-voxel resolution using phase contrast tomography. Tomogr Mater Struct. 2025;8:100060. doi: 10.1016/j.tmater.2025.100060

[162] Nolze G. Geometrically caused image distortion effects and their influence on interpretation of ebsd measurements. Mater Sci Technol. 2006;22(11):1343–1351. doi: 10.1179/174328406X130894

[163] Nolze G. Image distortions in sem and their influences on ebsd measurements. Ultramicroscopy. 2007;107(2–3):172–183. doi: 10.1016/j.ultramic.2006.07.003 17014961

[164] Charpagne MA, Strub F, Pollock TM. Accurate reconstruction of EBSD datasets by a multimodal data approach using an evolutionary algorithm. Mater Charact. 2019;150:184–198. doi: 10.1016/j.matchar.2019.01.033

[165] Tong VS, Ben BT. TrueEBSD: correcting spatial distortions in electron backscatter diffraction maps. Ultramicroscopy. 2021;221:113130. doi: 10.1016/j.ultramic.2020.113130 33290982

[166] Yin W, Briffod F, Hu H, et al. Role of prior austenite grain boundary and retained austenite in strain localization of medium-carbon high-strength steels. Acta Materialia. 2024;281:120422. doi: 10.1016/j.actamat.2024.120422

[167] Zhang Y, Elbrønd A, Lin F. A method to correct coordinate distortion in EBSD maps. Mater Charact. 2014;96:158–165. doi: 10.1016/j.matchar.2014.08.003

[168] Harte A, Atkinson M, Smith A, et al. The effect of solid solution and gamma prime on the deformation modes in Ni-based superalloys. Acta Materialia. 2020;194:257–275. doi: 10.1016/j.actamat.2020.04.004

[169] Idrissi Y, Richeton T, Texier D, et al. Robust determination of cubic elastic constants via nanoindentation and Bayesian inference. Acta Materialia. 2024;281:120406. doi: 10.1016/j.actamat.2024.120406

[170] Tasan C, Hoefnagels J, Diehl M, et al. Strain localization and damage in dual phase steels investigated by coupled in-situ deformation experiments and crystal plasticity simulations. Int J Plasticity. 2014;63:198–210. doi: 10.1016/j.ijplas.2014.06.004

[171] Tasan C, Diehl M, Yan D, et al. Integrated experimental–simulation analysis of stress and strain partitioning in multiphase alloys. Acta Materialia. 2014;81:386–400. doi: 10.1016/j.actamat.2014.07.071

[172] Bridier F, Villechaise P, Mendez J. Analysis of the different slip systems activated by tension in a alpha/beta titanium alloy in relation with local crystallographic orientation. Acta Materialia. 2005;53(3):555–567. doi: 10.1016/j.actamat.2004.09.040

[173] Wang XG, Witz JF, El Bartali A, et al. A dedicated dic methodology for characterizing plastic deformation in single crystals. Exp Mech. 2016;56(7):1155–1167. doi: 10.1007/s11340-016-0159-9

[174] Sperry R, Harte A, Quinta da Fonseca J, et al. Slip band characteristics in the presence of grain boundaries in nickel-based superalloy. Acta Materialia. 2020;193:229–238. doi: 10.1016/j.actamat.2020.04.037

[175] Baudoin P, Hama T, Takuda H. Influence of critical resolved shear stress ratios on the response of a commercially pure titanium oligocrystal: crystal plasticity simulations and experiment. Int J Plasticity. 2019;115:111–131. doi: 10.1016/j.ijplas.2018.11.013

[176] Vermeij T, Slokker G, Mornout CJA, et al. +sslip: automated radon-assisted and rotation-corrected identification of complex HCP slip system activity fields from DIC data. Strain. 2025;61(1). doi: 10.1111/str.70000

[177] Mornout C, Slokker G, Vermeij T, et al. Slide: automated identification and quantification of grain boundary sliding and opening in 3D. Scr Materialia. 2025;268:116861. doi: 10.1016/j.scriptamat.2025.116861

[178] Zhang M, Zhong L, Ju W, et al. Modeling hetero-deformation induced stress partitioning revealing non-basal slip activity in bimodal-grained ZK60 Mg alloy. J Magnesium Alloys. 2025;13(11):5745–5762. doi: 10.1016/j.jma.2025.05.003

[179] Linne MA, Bieler TR, Daly S. The effect of microstructure on the relationship between grain boundary sliding and slip transmission in high purity aluminum. Int J Plasticity. 2020;135:102818. doi: 10.1016/j.ijplas.2020.102818

[180] El Kadiri H, Kapil J, Oppedal A, et al. The effect of twin–twin interactions on the nucleation and propagation of {101¯2}{101¯2} twinning in magnesium. Acta Materialia. 2013;61(10):3549–3563. doi: 10.1016/j.actamat.2013.02.030

[181] Hazeli K, Askari H, Cuadra J, et al. Microstructure-sensitive investigation of magnesium alloy fatigue. Int J Plasticity. 2015;68:55–76. doi: 10.1016/j.ijplas.2014.10.010

[182] Abdolvand H, Wilkinson AJ. On the effects of reorientation and shear transfer during twin formation: comparison between high resolution electron backscatter diffraction experiments and a crystal plasticity finite element model. Int J Plasticity. 2016;84:160–182. doi: 10.1016/j.ijplas.2016.05.006

[183] Yaghoobi M, Chen Z, Sundararaghavan V, et al. Crystal plasticity finite element modeling of extension twinning in WE43 Mg alloys: calibration and validation. Integr Mater Manuf Innov. 2021;10(3):488–507. doi: 10.1007/s40192-021-00229-0

[184] Chen Z, Daly S. Deformation twin identification in magnesium through clustering and computer vision. Mater Sci Eng: A. 2018;736:61–75. doi: 10.1016/j.msea.2018.08.083

[185] Soleimani M, Kalhor A, Mirzadeh H. Transformation-induced plasticity (TRIP) in advanced steels: a review. Mater Sci Eng: A. 2020;795:140023. doi: 10.1016/j.msea.2020.140023

[186] Maresca F, Polatidis E, Šmíd M, et al. Measurement and prediction of the transformation strain that controls ductility and toughness in advanced steels. Acta Materialia. 2020;200:246–255. doi: 10.1016/j.actamat.2020.08.028

[187] Bieler T, Eisenlohr P, Zhang C, et al. Grain boundaries and interfaces in slip transfer. Curr Opin Solid State Mater Sci. 2014;18(4):212–226. doi: 10.1016/j.cossms.2014.05.003

[188] Bayerschen E, McBride AT, Reddy BD, et al. Review on slip transmission criteria in experiments and crystal plasticity models. J Mater Sci. 2015;51(5):2243–2258. doi: 10.1007/s10853-015-9553-4

[189] Hunter A, Leu B, Beyerlein IJ. A review of slip transfer: applications of mesoscale techniques. J Mater Sci. 2017;53(8):5584–5603. doi: 10.1007/s10853-017-1844-5

[190] Luster J, Morris MA. Compatibility of deformation in two-phase Ti-Al alloys: dependence on microstructure and orientation relationships. Metallurgical Mater Trans A. 1995;26(7):1745–1756. doi: 10.1007/BF02670762

[191] Seal J, Bieler T, Crimp M, et al. Characterizing slip transfer in commercially pure titanium using high resolution electron backscatter diffraction (HR-EBSD) and electron channeling contrast imaging (ECCI). Microsc Microanal. 2012;18(S2):702–703. doi: 10.1017/S1431927612005363

[192] Edwards TEJ, Di Gioacchino F, Clegg WJ. An experimental study of the polycrystalline plasticity of lamellar titanium aluminide. Int J Plasticity. 2019;118:291–319. doi: 10.1016/j.ijplas.2019.02.013

[193] Di Gioacchino F, Edwards TEJ, Wells GN, et al. A new mechanism of strain transfer in polycrystals. Sci Rep. 2020;10(1). doi: 10.1038/s41598-020-66569-7 PMC7308395 32572048

[194] Black RL, Garbowski T, Bean C, et al. High-throughput high-resolution digital image correlation measurements by multi-beam sem imaging. Exp Mech. 2023;63(5):939–953. doi: 10.1007/s11340-023-00961-y

[195] Bean C, Calvat M, Anjaria D, et al. Statistical analyses of plastic deformation events via computer vision: case study of additive manufactured microstructures. Mater Charact. 2025;228:115406. doi: 10.1016/j.matchar.2025.115406

[196] Hu D, Smith AD, Lunt D, et al. Tracking the onset of plasticity in a Ni-base superalloy using in-situ high-resolution digital image correlation. Mater Charact. 2025;220:114654. doi: 10.1016/j.matchar.2024.114654

[197] Yavuzyegit B, Karali K, Davis S, et al. High-resolution DIC analysis of in situ strain and crack propagation in coated AZ31 magnesium alloys under mechanical loading. J Mater Sci. 2025;60(33):14708–14730. doi: 10.1007/s10853-025-11243-4 40893464 PMC12397167

[198] Jia R, Zeng W, Zhao Z, et al. In situ EBSD/HR-DIC-based investigation on anisotropy mechanism of a near titanium plate with strong transverse texture. Mater Sci Eng: A. 2023;867:144743. doi: 10.1016/j.msea.2023.144743

[199] Li C, Jin J, Li X, et al. In situ dic and ebsd study of plasticity anisotropy, strain partitioning and strain localization in a duplex stainless steel. Mater Sci Eng: A. 2025;944:148874. doi: 10.1016/j.msea.2025.148874

[200] Smith AD, Lunt D, Taylor M, et al. A new approach to SEM *in-situ* thermomechanical experiments through automation. Ultramicroscopy. 2026;280:114244. Available from: https://www.sciencedirect.com/science/article/pii/S0304399125001421 41205358 10.1016/j.ultramic.2025.114244

[201] Stinville J, Callahan P, Charpagne M, et al. Direct measurements of slip irreversibility in a nickel-based superalloy using high resolution digital image correlation. Acta Materialia. 2020;186:172–189. doi: 10.1016/j.actamat.2019.12.009

[202] Kabel J, Edwards TE, Sharma A, et al. Direct observation of the elasticity-texture relationship in pyrolytic carbon via in situ micropillar compression and digital image correlation. Carbon. 2021;182:571–584. doi: 10.1016/j.carbon.2021.06.045

[203] Daum B, Dehm G, Clemens H, et al. Elastoplastic buckling as source of misinterpretation of micropillar tests. Acta Materialia. 2013;61(13):4996–5007. doi: 10.1016/j.actamat.2013.04.046

[204] de Barros Jo A, Schwiedrzik J, Wittel FK. Resolving discrepancies in wood micromechanics: strain-mapped compression of tracheid wall micropillars. Composites part a: applied science and manufacturing. Compos Part A Appl Sci Manuf. 2025;199:109209. doi: 10.1016/j.compositesa.2025.109209

[205] della Ventura NM, Sharma A, Cayron C, et al. Response of magnesium microcrystals to c-axis compression and contraction loadings at low and high strain rates. Acta Materialia. 2023;248:118762. doi: 10.1016/j.actamat.2023.118762

[206] Schaffer M, Schaffer B, Ramasse Q. Sample preparation for atomic-resolution STEM at low voltages by FIB. Ultramicroscopy. 2012;114:62–71. doi: 10.1016/j.ultramic.2012.01.005 22356790

[207] Macak EB, Münz WD, Rodenburg JM. Plasma–surface interaction at sharp edges and corners during ion-assisted physical vapor deposition. Part I: edge-related effects and their influence on coating morphology and composition. J Appl Phys. 2003;94(5):2829–2836. doi: 10.1063/1.1597755

[208] Edwards TEJ, Maeder X, Ast J, et al. Mapping pure plastic strains against locally applied stress: revealing toughening plasticity. Sci Adv. 2022;8(30). doi: 10.1126/sciadv.abo5735 PMC9328672 35895819

[209] Pürstl J, Jones H, Edwards T, et al. On the extraction of yield stresses from micro-compression experiments. Mater Sci Eng: A. 2021;800:140323. doi: 10.1016/j.msea.2020.140323

[210] Edwards TEJ, Di Gioacchino F, Goodfellow AJ, et al. Transverse deformation of a lamellar TiAl alloy at high temperature by in situ microcompression. Acta Materialia. 2019;166:85–99. doi: 10.1016/j.actamat.2018.11.050

[211] Edwards TEJ, Di Gioacchino F, Goodfellow AJ, et al. Deformation of lamellar -tial below the general yield stress. Acta Materialia. 2019;163:122–139. doi: 10.1016/j.actamat.2018.09.061

[212] Jones RD, Di Gioacchino F, Lim H, et al. Reduced partitioning of plastic strain for strong and yet ductile precipitate-strengthened alloys. Sci Rep. 2018;8(1). doi: 10.1038/s41598-018-26917-0 PMC5989271 29875381

[213] Lauener CM, Schwarz F, Pethö L, et al. Orientation-dependent extreme shear strain in single-crystalline silicon - from elasticity to fracture. Mater amp Des. 2023;235:112423. doi: 10.1016/j.matdes.2023.112423

[214] Alfreider M, Meindlhumer M, Maier-Kiener V, et al. Extracting information from noisy data: strain mapping during dynamic in situ SEM experiments. J Mater Res. 2021;36(11):2291–2304. doi: 10.1557/s43578-020-00041-0 34290473 PMC7611314

[215] Korsunsky AM, Sebastiani M, Bemporad E. Focused ion beam ring drilling for residual stress evaluation. Mater Lett. 2009;63(22):1961–1963. doi: 10.1016/j.matlet.2009.06.020

[216] Lord J, Cox D, Ratzke A, et al. A good practice guide for measuring residual stresses using FIB-DIC. National Physical Laboratory (NPL). Measurement Good Practice Guide 143. Available from 2018. Available from: https://eprintspublications.npl.co.uk/id/eprint/7807

[217] Wouters O, Vellinga W, Tijum RV, et al. On the evolution of surface roughness during deformation of polycrystalline aluminum alloys. Acta Materialia. 2005;53(15):4043–4050. doi: 10.1016/j.actamat.2005.05.007

[218] Lackmann J, Niendorf T, Maxisch M, et al. High-resolution in-situ characterization of the surface evolution of a polycrystalline NiTi SMA-alloy under pseudoelastic deformation. Mater Charact. 2011;62(3):298–303. doi: 10.1016/j.matchar.2010.12.008

[219] Bertin M, Du C, Hoefnagels JP, et al. Crystal plasticity parameter identification with 3d measurements and integrated digital image correlation. Acta Materialia. 2016;116:321–331. doi: 10.1016/j.actamat.2016.06.039

[220] Liu J, Vanderesse N, Stinville JC, et al. In-plane and out-of-plane deformation at the sub-grain scale in polycrystalline materials assessed by confocal microscopy. Acta Materialia. 2019;169:260–274. doi: 10.1016/j.actamat.2019.03.001

[221] Britton T, Wilkinson A. Measurement of residual elastic strain and lattice rotations with high resolution electron backscatter diffraction. Ultramicroscopy. 2011;111(8):1395–1404. doi: 10.1016/j.ultramic.2011.05.007 21864783

[222] Britton TB, Jiang J, Karamched PS, et al. Probing deformation and revealing microstructural mechanisms with cross-correlation-based, high-resolution electron backscatter diffraction. JOM. 2013;65(9):1245–1253. doi: 10.1007/s11837-013-0680-6

[223] Britton TB, Hickey JLR. Understanding deformation with high angular resolution electron backscatter diffraction (HR-EBSD). In: IOP Conference Series: Materials Science and Engineering; Konstanz, Germany; 2018; Vol304. p. 012003. doi: 10.1088/1757-899X/304/1/012003

[224] Jiang J, Britton TB, Wilkinson AJ. Mapping type III intragranular residual stress distributions in deformed copper polycrystals. Acta Materialia. 2013;61(15):5895–5904. doi: 10.1016/j.actamat.2013.06.038

[225] Zhang T, Jiang J, Shollock BA, et al. Slip localization and fatigue crack nucleation near a non-metallic inclusion in polycrystalline nickel-based superalloy. Mater Sci Eng: A. 2015;641:328–339. doi: 10.1016/j.msea.2015.06.070

[226] Zhang T, Jiang J, Britton B, et al. Crack nucleation using combined crystal plasticity modelling, high-resolution digital image correlation and high-resolution electron backscatter diffraction in a superalloy containing non-metallic inclusions under fatigue. In: Proceedings of the Royal Society A: Mathematical, Physical and Engineering Sciences; 2016; Vol472. p. 20150792. doi: 10.1098/rspa.2015.0792 PMC4893176 27279765

[227] Zhao C, Stewart D, Jiang J, et al. A comparative assessment of iron and cobalt-based hard-facing alloy deformation using HR-EBSD and HR-DIC. Acta Materialia. 2018;159:173–186. doi: 10.1016/j.actamat.2018.08.021

[228] Yamasaki S, Matsuo H, Morikawa T, et al. Acquisition of microscopic and local stress-strain curves by combination of HR-EBSD and DIC methods. Scr Materialia. 2023;235:115603. doi: 10.1016/j.scriptamat.2023.115603

[229] Ueno K, Hiwatashi S, Sakaguchi K, et al. Microstructural stress-strain analysis in lath martensitic steel: insights into slip system activity. Mater Today Commun. 2025;44:112005. doi: 10.1016/j.mtcomm.2025.112005

[230] Britton T, Maurice C, Fortunier R, et al. Factors affecting the accuracy of high resolution electron backscatter diffraction when using simulated patterns. Ultramicroscopy. 2010;110(12):1443–1453. doi: 10.1016/j.ultramic.2010.08.001 20888125

[231] Fullwood D, Vaudin M, Daniels C, et al. Validation of kinematically simulated pattern HR-EBSD for measuring absolute strains and lattice tetragonality. Mater Charact. 2015;107:270–277. doi: 10.1016/j.matchar.2015.07.017

[232] Vermeij T, De Graef M, Hoefnagels J. Demonstrating the potential of accurate absolute cross-grain stress and orientation correlation using electron backscatter diffraction. Scr Materialia. 2019;162:266–271. doi: 10.1016/j.scriptamat.2018.11.030

[233] Shi Q, Zhong H, Loisnard D, et al. Towards measuring absolute residual stress by HR-EBSD with simulated reference patterns. Mater Charact. 2024;218:114508. doi: 10.1016/j.matchar.2024.114508

[234] Roters F, Eisenlohr P, Hantcherli L, et al. Overview of constitutive laws, kinematics, homogenization and multiscale methods in crystal plasticity finite-element modeling: theory, experiments, applications. Acta Materialia. 2010;58(4):1152–1211. doi: 10.1016/j.actamat.2009.10.058

[235] Roters F, Diehl M, Shanthraj P, et al. Damask – the Düsseldorf advanced material simulation kit for modeling multi-physics crystal plasticity, thermal, and damage phenomena from the single crystal up to the component scale. Comput Mater Sci. 2019;158:420–478. doi: 10.1016/j.commatsci.2018.04.030

[236] Yaghoobi M, Ganesan S, Sundar S, et al. Prisms-plasticity: an open-source crystal plasticity finite element software. Comput Mater Sci. 2019;169:109078. doi: 10.1016/j.commatsci.2019.109078

[237] Lee EH, Liu DT. Finite-strain elastic—plastic theory with application to plane-wave analysis. J Appl Phys. 1967;38(1):19–27. doi: 10.1063/1.1708953

[238] Chaboche J. A review of some plasticity and viscoplasticity constitutive theories. Int J Plasticity. 2008;24(10):1642–1693. doi: 10.1016/j.ijplas.2008.03.009

[239] Marano A, Gélébart L, Forest S. Intragranular localization induced by softening crystal plasticity: analysis of slip and kink bands localization modes from high resolution FFT-simulations results. Acta Materialia. 2019;175:262–275. doi: 10.1016/j.actamat.2019.06.010

[240] Marano A, Gélébart L, Forest S. Fft-based simulations of slip and kink bands formation in 3d polycrystals: influence of strain gradient crystal plasticity. J The Mech Phys Solids. 2021;149:104295. doi: 10.1016/j.jmps.2021.104295

[241] Vermeij T, Wijnen J, Peerlings R, et al. A quasi-2D integrated experimental–numerical approach to high-fidelity mechanical analysis of metallic microstructures. Acta Materialia. 2024;264:119551. doi: 10.1016/j.actamat.2023.119551

[242] Diehl M, Shanthraj P, Eisenlohr P, et al. Neighborhood influences on stress and strain partitioning in dual-phase microstructures: an investigation on synthetic polycrystals with a robust spectral-based numerical method. Meccanica. 2015;51(2):429–441. doi: 10.1007/s11012-015-0281-2

[243] Diehl M, An D, Shanthraj P, et al. Crystal plasticity study on stress and strain partitioning in a measured 3d dual phase steel microstructure. Phys Mesomech. 2017;20(3):311–323. doi: 10.1134/S1029959917030079

[244] Wijnen J, Vermeij T, Hoefnagels J, et al. High-resolution numerical–experimental comparison of heterogeneous slip activity in quasi-2d ferrite sheets. Int J Solids Struct. 2025;320:113523. doi: 10.1016/j.ijsolstr.2025.113523

[245] Echlin MP, Straw M, Randolph S, et al. The tribeam system: femtosecond laser ablation in situ SEM. Mater Charact. 2015;100:1–12. doi: 10.1016/j.matchar.2014.10.023

[246] Echlin MP, Stinville JC, Miller VM, et al. Incipient slip and long range plastic strain localization in microtextured Ti-6Al-4V titanium. Acta Materialia. 2016;114:164–175. doi: 10.1016/j.actamat.2016.04.057

[247] Echlin MP, Burnett TL, Polonsky AT, et al. Serial sectioning in the SEM for three dimensional materials science. Curr Opin Solid State Mater Sci. 2020;24(2):100817. doi: 10.1016/j.cossms.2020.100817

[248] Shi Q, Latourte F, Hild F, et al. Backtracking depth-resolved microstructures for crystal plasticity identification—part 2: identification. JOM. 2017;69(12):2803–2809. doi: 10.1007/s11837-017-2586-1

[249] Marano A, Ribart C, Proudhon H. Towards a data platform for multimodal 4D mechanics of material microstructures. Mater amp Des. 2024;246:113306. doi: 10.1016/j.matdes.2024.113306

[250] Mesbah D, Proudhon H, Gélébart L, et al. Multimodal super-resolution for fast image-based simulation of crystal plasticity. Comput Methods Appl Mech Eng. 2025;445:118210. doi: 10.1016/j.cma.2025.118210

[251] Quey R, Dawson P, Barbe F. Large-scale 3D random polycrystals for the finite element method: generation, meshing and remeshing. Comput Methods Appl Mech Eng. 2011;200(17–20):1729–1745. doi: 10.1016/j.cma.2011.01.002

[252] Groeber MA, Ma J. Dream.3D: a digital representation environment for the analysis of microstructure in 3D. Integr Mater Manuf Innov. 2014;3(1):56–72. doi: 10.1186/2193-9772-3-5

[253] Depriester D, Kubler R. Mtex2gmsh: a tool for generating 2d meshes from EBSD data. J Open Source Softw. 2020;5(52):2094. doi: 10.21105/joss.02094

[254] Hestroffer JM, Latypov MI, Stinville JC, et al. Development of grain-scale slip activity and lattice rotation fields in Inconel 718. Acta Materialia. 2022;226:117627. doi: 10.1016/j.actamat.2022.117627

[255] Zhu J, Briffod F, Shiraiwa T, et al. $ two-phase ti–12mo alloy under compressive condition. Mater Trans. 2023;64(12):2677–2686. doi: 10.2320/matertrans.MT-M2023111

[256] Briffod F, Shen Y, Hu H, et al. Effect of microstructure on the deformation of as-cast: an integrated SEM-DIC and crystal plasticity study. Materialia. 2024;33:102015. doi: 10.1016/j.mtla.2024.102015

[257] Ruybalid A, Hoefnagels J, van der Sluis O, et al. Mixed-mode cohesive zone parameters from integrated digital image correlation on micrographs only. Int J Solids Struct. 2019;156–157:179–193. doi: 10.1016/j.ijsolstr.2018.08.010

[258] Depriester D, Goulmy J, Barrallier L. Crystal plasticity simulations of in situ tensile tests: a two-step inverse method for identification of cp parameters, and assessment of cpfem capabilities. Int J Plasticity. 2023;168:103695. doi: 10.1016/j.ijplas.2023.103695

[259] Rokoš O, Hoefnagels J, Peerlings R, et al. On micromechanical parameter identification with integrated DIC and the role of accuracy in kinematic boundary conditions. Int J Solids Struct. 2018;146:241–259. doi: 10.1016/j.ijsolstr.2018.04.004

[260] Githens A, Ganesan S, Chen Z, et al. Characterizing microscale deformation mechanisms and macroscopic tensile properties of a high strength magnesium rare-earth alloy: a combined experimental and crystal plasticity approach. Acta Materialia. 2020;186:77–94. doi: 10.1016/j.actamat.2019.12.012

[261] Chen B, Jiang J, Dunne FP. Is stored energy density the primary meso-scale mechanistic driver for fatigue crack nucleation? Int J Plasticity. 2018;101:213–229. doi: 10.1016/j.ijplas.2017.11.005

[262] Latypov MI, Stinville JC, Mayeur JR, et al. Insight into microstructure-sensitive elastic strain concentrations from integrated computational modeling and digital image correlation. Scr Materialia. 2021;192:78–82. doi: 10.1016/j.scriptamat.2020.10.001

[263] Zhang X, Stinville JC, Pollock TM, et al. Crystallography and elastic anisotropy in fatigue crack nucleation at nickel alloy twin boundaries. J The Mech Phys Solids. 2021;155:104538. doi: 10.1016/j.jmps.2021.104538

[264] Zhang M, Bridier F, Villechaise P, et al. Simulation of slip band evolution in duplex Ti–6Al–4V. Acta Materialia. 2010;58(3):1087–1096. doi: 10.1016/j.actamat.2009.10.025

[265] Wijnen J, Peerlings R, Hoefnagels J, et al. A discrete slip plane model for simulating heterogeneous plastic deformation in single crystals. Int J Solids Struct. 2021;228:111094. doi: 10.1016/j.ijsolstr.2021.111094

[266] Ahmadikia B, Bean C, Stinville JC, et al. Modeling the evolution of slip localization: realization of link to material strength. Acta Materialia. 2025;299:121314. doi: 10.1016/j.actamat.2025.121314

[267] Besson J. Continuum models of ductile fracture: a review. Int J Damage Mech. 2009;19(1):3–52. doi: 10.1177/1056789509103482

[268] Bean C, Calvat M, Nie Y, et al. Accelerated fatigue strength prediction via additive manufactured functionally graded materials and high-throughput plasticity quantification. Mater amp Des. 2025;256:114115. doi: 10.1016/j.matdes.2025.114115

[269] Calvat M, Bean C, Anjaria D, et al. Plasticity encoding and mapping during elementary loading for accelerated mechanical properties prediction. Scr Materialia. 2026;273:117082. doi: 10.1016/j.scriptamat.2025.117082

[270] Vieira RB, Lambros J. Machine learning neural-network predictions for grain-boundary strain accumulation in a polycrystalline metal. Exp Mech. 2021;61(4):627–639. doi: 10.1007/s11340-020-00687-1

[271] Hardie C, Thomas R, Liu Y, et al. Simulation of crystal plasticity in irradiated metals: a case study on zircaloy-4. Acta Materialia. 2022;241:118361. https://www.sciencedirect.com/science/article/pii/S1359645422007406

[272] Mizuno Y, Hosoi A, Koshita H, et al. Fatigue life prediction of composite materials using strain distribution images and a deep convolution neural network. Sci Rep. 2024;14(1). doi: 10.1038/s41598-024-75884-2 PMC11511892 39455711

[273] Kiu MF, Pinna C, Farrugia DCJ. New experimental procedure for the analysis of micro-scale surface damage at high temperature. Exp Mech. 2016;56(6):1063–1072. doi: 10.1007/s11340-016-0151-4

[274] Edwards TEJ, Di Gioacchino F, Clegg WJ. High resolution digital image correlation mapping of strain localization upon room and high temperature, high cycle fatigue of a TiAl intermetallic alloy. Int J Fatigue. 2021;142:105905. doi: 10.1016/j.ijfatigue.2020.105905

[275] Roy R, Topping M, Long F, et al. Effect of irradiation-induced microstructure on dislocation channeling and strain localization in Zircaloy-4. Acta Materialia. 2025;300:121490. doi: 10.1016/j.actamat.2025.121490

[276] Calvat M, Bean C, Anjaria D, et al. Learning metal microstructural heterogeneity through spatial mapping of diffraction latent space features. Npj Comput Mater. 2025;11(1). doi: 10.1038/s41524-025-01770-8

[277] Wu H, Fan G. An overview of tailoring strain delocalization for strength-ductility synergy. Prog Mater Sci. 2020;113:100675. doi: 10.1016/j.pmatsci.2020.100675

[278] Ahmadikia B, Beyerlein AL, Hestroffer JM, et al. Designing Ti-6Al-4V microstructure for strain delocalization using neural networks. J Mater Sci Mater Theory. 2024;8(1). doi: 10.1186/s41313-024-00055-9

